# Temperature–Photoperiod Interaction in Rice Phenology for Climate Adaptation: Insights Into Glycerate‐Associated Metabolic Responses

**DOI:** 10.1111/ppl.70368

**Published:** 2025-07-08

**Authors:** Hyeon‐Seok Lee, Ju‐Hee Kim, So‐Hye Jo, Seo‐Yeong Yang, Jae‐Kyeong Baek, Yeong‐Seo Song, Ji‐young Shon, Nam‐Jin Chung

**Affiliations:** ^1^ Crop Production & Physiology Division National Institute of Crop Science, Rural Development Administration Wanju‐Gun Republic of Korea; ^2^ Department of Crop Science and Biotechnology Chonbuk National University Jeonju Republic of Korea

**Keywords:** glycerate, heading, photosensitivity, rice, thermosensitivity

## Abstract

Rice heading date is tightly regulated by photoperiod and temperature, which are critical environmental cues for climate adaptation. While photoperiodic control of flowering has been well characterized, the molecular and metabolic mechanisms underlying temperature responses and their interaction with photoperiod remain unclear. In this study, we used two *Oryza sativa* ssp. japonica cultivars under controlled conditions to investigate the effects of temperature (22°C vs. 28°C) and photoperiod (12 vs. 14.5 h) during the photo‐sensitive period. Integrative transcriptomic and metabolomic analyses identified key regulators of heading time, with particular focus on glycerate metabolism. Thermosensitivity increased threefold under short‐day conditions, while photosensitivity was enhanced under high temperature. Glycerate, a pivotal intermediate in photorespiration and glycolysis, showed an inverse correlation with days to heading and accumulated more strongly in leaves under short‐day and high‐temperature conditions. Exogenous glycerate application (250–500 μM) accelerated heading by 4–5 days, supporting its functional role in floral induction. These findings highlight glycerate‐ and serine‐associated metabolic pathways in regulating heading responses to environmental cues, providing new perspectives for optimizing heading time and enhancing climate resilience in rice production.

## 1 Introduction

Climate change significantly alters the environmental conditions under which crops are cultivated, leading to unpredictable shifts in crop phenological timing (Lee et al. 2011). In rice, heading date determines the duration of both vegetative and reproductive growth phases (Cui et al. 2019; Liu et al. 2014; Mo et al. 2016), which in turn affect grain filling conditions, ultimately impacting yield quantity and quality. Consequently, optimizing heading time is crucial for climate‐resilient rice production strategies (Lee, Choi, et al. 2019; Xu et al. 2021).

While both photoperiod and temperature are key regulators of heading time in rice, their respective contributions differ in the context of climate variability (Nishida et al. 2001; Komiya et al. 2009; Kovi et al. 2013). Photoperiod is governed by stable astronomical cycles and remains relatively constant year to year, whereas temperature exhibits high interannual and regional variability due to climate change (Horie 2019). This distinction implies that temperature–photoperiod interactions must be better understood to predict and manage phenological development under future environmental conditions.

Photoperiodic control of flowering has been well studied in rice (Jian et al. 2021; Wu et al. 2020). Under short‐day conditions, rice activates florigen genes such as *Hd3a* and *RFT1*, which induce panicle initiation (Tamaki et al. 2007; Tsuji et al. 2011). These florigen genes integrate environmental signals through upstream regulators, including photoreceptors (e.g., *PhyA*, *PhyB*, *CRY1*) and circadian clock components (e.g., *OsTOC1*, *OsCCA1*) (Lee and An 2015; Shen et al. 2015). In contrast, relatively little is known about how temperature affects heading in rice at the molecular level, despite its strong influence on phenological timing (Nagalla et al. 2021; Zong et al. 2024).

Unlike photoperiod, which follows predictable seasonal cycles governed by Milankovitch patterns, temperature can fluctuate dramatically, even under consistent agronomic practices such as fixed transplanting dates (Ishigooka 2017; Lee et al. 2011). Accordingly, understanding how temperature modifies heading responses—and how it interacts with photoperiod—is essential for refining crop models and management practices for climate‐resilient rice production (Lee et al. 2021).

Much of our mechanistic understanding of temperature‐dependent flowering comes from studies in *Arabidopsis thaliana* . Qiu et al. (2019) demonstrated that daytime temperature sensing is mediated by phytochrome B (*phyB*) via *PIF4* and *HEMERA*, while Zhou et al. (2024) showed that *phyB* and *CRY1* act cooperatively to regulate thermoresponsive flowering through modulation of *PIF4*. These findings illustrate how plants integrate light and temperature signals to control development. However, such detailed molecular insights remain scarce in rice (Nagalla et al. 2021; Zong et al. 2024).

While direct regulatory pathways are not yet fully elucidated, several studies have explored the temperature influence on flowering and heading time in rice. Specifically, stage‐specific temperature sensitivity has been characterized, indicating that the responsiveness to temperature varies across developmental stages (Lee, Hwang, et al. 2019; Yin et al. 1997). Furthermore, the modulation of florigen gene expression by environmental factors such as temperature and photoperiod has been examined. For instance, *Hd3a* expression is reportedly suppressed under low temperatures regardless of photoperiod (Luan et al. 2009), whereas the *Hd1–Ghd7–Ehd1–RFT1* regulatory module is repressed by low temperatures specifically under long‐day conditions (Song and Luan 2012).

Concurrently, although photoperiodic control of flowering has been extensively characterized, the role of fundamental metabolic processes—including photosynthesis, respiration, and metabolic fluctuations—in regulating heading time remains insufficiently understood (Cho et al. 2018; Kim et al. 2025). Notably, emerging research emphasizes that metabolites function not only as substrates for growth but also as integrators of environmental cues that coordinate developmental transitions such as flowering (Borghi et al. 2019). Adjustments in central metabolic pathways, including sugar and amino acid metabolism, are increasingly recognized as crucial modulators of floral development in response to climatic variability (Borghi et al. 2019). Nevertheless, the contributions of other metabolites to heading regulation in rice remain largely unexplored.

In this study, we hypothesized that environmental changes in temperature and photoperiod would induce coordinated shifts in metabolic profiles and gene expression related to heading regulation. We conducted an integrative phenotypic, transcriptomic, and metabolomic analysis under defined combinations of temperature and daylength to identify specific genes and metabolites associated with heading responses. To test this hypothesis, we conducted a controlled‐environment experiment integrating phenotypic, transcriptomic, and metabolomic analyses. Rice plants were exposed to four combinations of temperature and photoperiod during the photo‐sensitive period (PSP), using two japonica cultivars with contrasting maturation types. This design enabled us to evaluate environmentally induced shifts in heading‐related gene expression and metabolism.

Collectively, this study provides new insights into how temperature and photoperiod interact with metabolic networks to regulate heading time in rice. These findings enhance our understanding of the physiological and metabolic mechanisms underlying rice adaptation to changing climatic conditions.

## 2 Materials and Methods

### 2.1 Ethics Statement

This study was performed in accordance with Institute‐approved guidelines and regulations. The test varieties were provided by SeoYeong Yang of the Rice Production and Physiology Division of the National Institute of Crop Science (NICS). Permission from the NICS was obtained to use these varieties (https://www.nics.go.kr/apo/breed.do?m=100000128&homepageSecod=nics).

### 2.2 Experimental Materials and Design

Two experiments were conducted in a controlled environment facility (ENT Inc., Boocheon, South Korea) at the National Institute of Crop Science in Jeonju, South Korea (35°49′19″ N, 127°8′56″ E), where light intensity, temperature, and humidity can be artificially controlled (Figure S1). Lights were turned on at 07:30 regardless of daylength treatment; daylength was adjusted by the lights‐off time. The light intensity was set at 800 μmol m^−2^ s^−1^ photosynthetically active radiation based on clear‐day conditions (Choi et al. 2013).

Two rice cultivars representing different maturation ecotypes were used: early‐maturing “Odae” ( *Oryza sativa* ssp. *japonica*, IT218242) and mid‐late‐maturing “Saenuri” ( *Oryza sativa* ssp. *japonica*, IT235281). These varieties were genetically analyzed by the Breeding Division of the National Institute of Crop Science (Lee et al. 2023) and are frequently utilized in phenology‐related research as representative cultivars of their ecotypes (Lee, Hwang, et al. 2019; Lee et al. 2021).

A composite slow‐release fertilizer based on 9, 4.5, and 5.7 kg/10 a of nitrogen, phosphate, and potassium, respectively, was used at an area ratio corresponding to three plants (planting distance: 30 × 14 cm) instead of the pot area. In all cultivars, 20‐day‐old seedlings were transplanted into 1/5000 Wagner pots at a density of three plants per pot.

#### 2.2.1 Experiment 1: Controlled‐Environment Study on Temperature and Photoperiod Effects in Rice

A controlled‐environment experiment was conducted using a completely randomized design, with temperature and photoperiod as independent factors: short‐day versus long‐day, low‐temperature versus high‐temperature. The treatment period was determined based on the rice growth stages that exhibit sensitivity to photoperiodic responses to ensure the most relevant experimental heading regulation conditions.

Rice growth before heading is divided into the basic vegetative period (BVP), the PSP, and the post‐photo‐sensitive period (PPP) (Yin et al. 1997). BVP and PPP are primarily influenced by temperature, whereas PSP is affected by photoperiod and temperature, with photoperiod sensitivity exclusive to PSP (Yin et al. 1997). The PPP corresponds to the panicle initiation stage, ~30–32 days before heading, irrespective of cultivar (Ahn and Vergara 1969; Yin et al. 1997).

**FIGURE 1:**
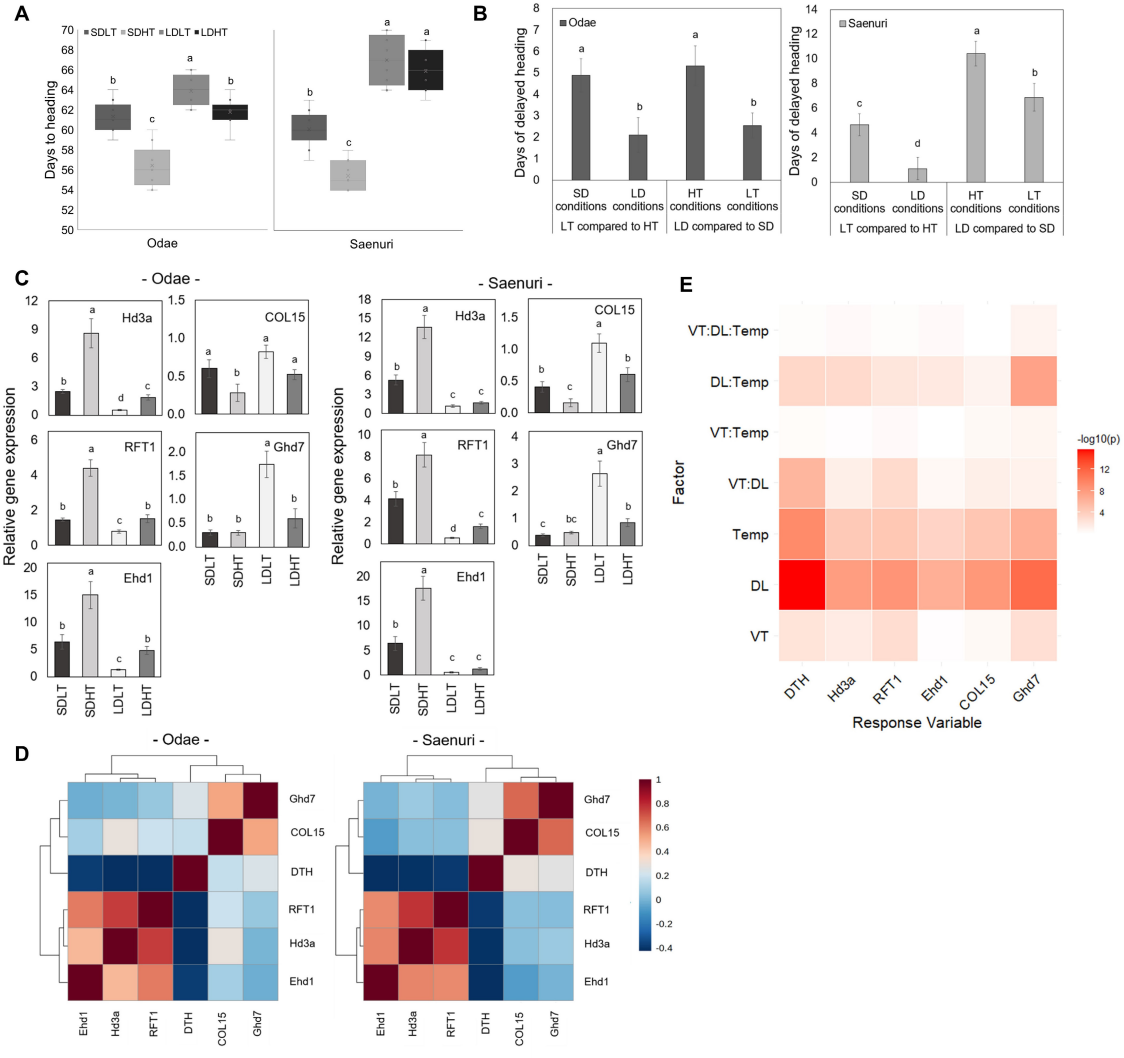
Effects of daylength and temperature on heading response and the expression of flowering‐related genes in rice. (A) Box plot showing variations in days to heading (DTH) under different temperature and daylength conditions. The black horizontal lines indicate the median, and the “×” symbol represents the mean. (B) Interaction effects of daylength and temperature on heading delay. (C) Relative expression levels of flowering‐related genes (Hd3a, RFT1, Ehd1, COL15, and Ghd7) under varying treatment conditions. (D) Correlation analysis between DTH and gene expression. (E) ANOVA‐based heatmap illustrating the effects of variety (VT), daylength (DL), and temperature (Temp) on DTH and gene expression. The color gradient represents –log_10_ (*p* value), with darker red indicating stronger statistical significance. Different letters indicate significant differences within each dataset based on Duncan's multiple range test (*p* < 0.05). DTH data were obtained from nine biological replicates per treatment (*n* = 9), while gene expression analyses were based on three biological replicates per condition, where samples were collected at five different time points per treatment. The mean expression value across the time series was used for statistical comparisons (*n* = 5).

Twenty‐day‐old seedlings were transplanted and given a 10‐day recovery period to mitigate transplant shock before treatment onset (Lee et al. 2021). The temperature and photoperiod treatments were applied for 12 days based on previous studies on the heading dates of Odae and Saenuri cultivars (Lee, Hwang, et al. 2019). The treatments were designed to coincide with PSP. The treatment periods were determined based on the earliest heading treatment group (Figure 1A). The treatment in Odae corresponded to 46 to 34 days before heading, and in Saenuri, 45–33 days before heading. This approach ensured that treatments were applied specifically during PSP, avoiding potential confounding effects on BVP and PPP.

The base temperature for rice growth is reported to be 8°C–12°C, with an optimal range of 25°C–30°C. Exposure to temperatures exceeding 35°C negatively affects growth due to heat stress (Krishnan et al. 2011). To maintain stable conditions before and after treatment, the control temperature was set at 25°C. Temperature treatments were applied during the 12‐day treatment period (low‐temperature: 22°C, maximum 27°C, minimum 17°C; high‐temperature: 28°C, maximum 33°C, minimum 23°C). During the pre‐treatment and post‐treatment (until heading) periods, the temperature was maintained at 25°C (maximum: 30°C, minimum: 20°C).

Before treatment initiation, the photoperiod was maintained at 15 h light/9 h dark to minimize pre‐treatment photoperiodic effects (Lee, Hwang, et al. 2019). During the 12‐day treatment period, short‐day conditions were maintained at 12 h light/12 h dark and long‐day conditions at 14 h 30 min light/9 h 30 min dark. These photoperiod conditions reflect natural daylength conditions in rice‐growing regions and are standard experimental settings for photoperiod research in rice (Nagalla et al. 2021; Song and Luan 2012). Following the treatment period, photoperiod conditions were reverted to 12 h light/12 h dark—the optimal condition for inducing photoperiodic sensitivity in the two cultivars used (Lee, Hwang, et al. 2019). This minimized the influence of further day‐length variations after the 12‐day treatment. A summary of the temperature–photoperiod treatment combinations is provided in Table 1

**TABLE 1:** Summary of the temperature and photoperiod treatments applied during the photo‐sensitive period (PSP) in the controlled‐environment experiment.

Abbreviation	Photoperiod condition	Light/dark cycle (h)	Temperature condition	Temperature range (°C)
SDLT	Short‐day	12/12	Low temperature	22 (17–27)
SDHT	Short‐day	12/12	High temperature	28 (23–33)
LDLT	Long‐day	14.5/9.5	Low temperature	22 (17–27)
LDHT	Long‐day	14.5/9.5	High temperature	28 (23–33)

*Note:* Photoperiod and temperature were manipulated independently to simulate short‐day (SD) and long‐day (LD) conditions, as well as low‐ (LT) and high‐temperature (HT) regimes.

Systematic sampling and growth measurements were conducted to assess plant responses at multiple time points. Data were collected one day before treatment initiation and 1, 2, 4, 7, and 10 days after treatment to evaluate growth responses and analyze transcriptomic and metabolomic changes.

#### 2.2.2 Experiment 2: Effects of Exogenous Glycerate Treatment on Growth and Heading Date

The glycerate treatment was conducted at different concentrations under conditions similar to Experiment 1. Twenty‐day‐old seedlings were transplanted, and treatments were initiated 10 days after transplantation, following the same pre‐treatment conditions as Experiment 1 to ensure that the treatment was applied after the BVP and during the PSP. After treatment initiation, the experiment was conducted under constant short‐day (12 h light/12 h dark) conditions at 21°C (maximum 26°C, minimum 16°C) until heading to evaluate the effects of glycerate treatment under controlled conditions.

D‐Glycerate (D‐glyceric acid sodium salt, ≥ 95.0% (TLC); Sigma‐Aldrich) was dissolved in distilled water supplemented with 0.01% (v/v) Tween 20 to enhance foliar absorption. Treatment concentrations were set at 62.5 , 125 , 250 , 500 , and 1000 μM. These concentrations were selected based on previous studies involving the exogenous application of organic acids to plants, where similar ranges (typically 0.1–1.0 mM) have been reported to induce physiological and metabolic responses without phytotoxicity. Notable examples include foliar treatments with citric acid, malic acid, and other organic acids, which demonstrated efficacy at comparable concentrations under stress conditions (Hu et al. 2021; Hei et al. 2024).

Each plant received approximately 3 mL of the solution, which was uniformly applied to the aerial parts using a fine mist sprayer. Treatments were administered twice per week at 10:00 a.m. for a total duration of 3 weeks, with 3–4 days between applications. Glycerate treatments were applied from 10 to 31 days after transplantation. Based on the heading dates under different temperature conditions, this period corresponded to approximately 51–30 days before heading for Odae at 21°C, 33–12 days at 24°C, and 27–23 days at 27°C; and for Saenuri, 58–37 days at 21°C, 35–14 days at 24°C, and 29–8 days at 27°C. This treatment schedule was designed to align with the PSP period as closely as possible. However, under higher temperature conditions, accelerated development likely compressed the PSP period, potentially limiting the effective exposure period to glycerate treatment.

Following the treatments, heading date was recorded, and heading‐related growth parameters, including panicle number per hill, spikelet number per panicle, panicle length, and culm length, were measured.

### 2.3 Growth, Development Factors, and Heading Date

Plant length was measured as the distance from the ground to the tip of the highest leaf. Culm length was determined as the distance from the ground to the uppermost internode, while panicle length was measured from the uppermost internode to the tip of the panicle. Panicle number was recorded on a per‐hill basis, whereas spikelet number was measured per panicle. Days to heading was defined as the number of days after transplanting until the panicle emerged from the leaf sheath. Panicle emergence was observed daily between 13:00 and 14:00 to ensure accurate determination of heading time.

### 2.4 Sampling Locations in Rice Plants for Metabolite and Transcriptome Analysis

Figure S2 displays the rice plant sampling locations used for transcriptome and metabolite analysis. Sampling was conducted at four locations: leaf, tiller, and stem (upper, lower). Plants from 1 day before treatment (Control) and 2 days after treatment were sampled.

### 2.5 Sample Preparation for Metabolomic Analysis

Each lyophilized sample was homogenized with 80% methanol using a bullet blender (Next Advance). After centrifugation, the supernatant was dried using a CentriVap concentrator SpeedVac (Labconco Co.). For gas chromatography–mass spectrometry (GC–MS) analysis, all dried samples were dissolved in 70 μL of methoxyamine hydrochloride in pyridine (20 mg/mL) containing dicyclohexyl‐phthalate as an internal standard and incubated at 37°C for 90 min. The methoxylated samples were derivatized using 70 μL N,O‐bis(trimethylsilyl)trifluoroacetamide with 1% trimethylchlorosilane at 70°C for 30 min. The derivatized samples were analyzed by GC–MS (Shimadzu Corp.) as previously described (Kim et al. 2020).

### 2.6 Analysis of Metabolites Using GC–MS

The plant extracts were analyzed using a GC‐2010 plus system (Shimadzu Corp.) equipped with a DB‐5 ms capillary column (30 m × 0.25 mm, 0.25 μm, Agilent J&W column, Agilent Technologies). The derivatized samples were injected into the column at a split ratio of 40. Helium was injected as a carrier gas with a flow rate of 1 mL/min at 200°C. The oven temperature was maintained at 70°C for 2 min, then increased at a rate of 10°C/min to 320°C and maintained for 5 min. The eluents were detected using a GCMS‐TQ‐8030‐MS (Shimadzu) system with electron ionization at 70 eV, an ion source temperature of 230°C, and an interface temperature of 280°C. The data were monitored and collected in the full‐scan mode in the mass range of m/z 45–550 with a scan event time of 0.3 s and a scan rate of 2000 amu/s. The QC sample was analyzed once for every five samples per a previously described protocol (Kim et al. 2020).

### 2.7 Data Processing for Metabolomic Analysis

Metabolite levels were determined by GC–MS and expressed as normalized intensity values. Absolute concentrations were not calculated due to the absence of internal standards; however, relative differences among treatment groups were used to assess metabolic responses.

The collection, normalization, and alignment of the MS dataset analyzed using the UPLC‐Q‐TOF MS were conducted using UNIFI version 1.8.2 (Waters Corp.). The GC–MS data were analyzed using the Analyzer Pro application (Spectralworks Ltd.). All data were normalized using the average mass intensity of the internal standards. The metabolites were identified based on the online databases (NIST‐11 and Wiley‐9 mass spectral libraries for GC–MS; ChemSpider database in UNIFI, METLIN database www.metlin.scripps.edu), and retention indices (RIs) were calculated using n‐alkanes for GC–MS. The SIMCA‐*P*+ version 12.0.1 (Umetrics) was used to analyze the GC–MS data. The values used in the heatmap analysis were normalized using Z‐score transformation in MetaboAnalyst as previously described (Kim et al. 2020).

### 2.8 RNA Extraction and Gene Expression

For RNA expression analysis, the 2nd and 3rd leaves of the main stem and tiller were collected from three individual plants, frozen in liquid nitrogen, and stored at −80°C. The second and third leaves were selected as they exhibit a more stable physiological response compared to the first leaf, which is still developing. Three biological replicates were conducted per treatment, with each replicate consisting of three plants. Plant material for RNA extraction was sampled at 10:00 (2.5 h after light exposure), as *Hd3a*, *RFT1*, *Ehd1*, and *Ghd7* maintain high expression levels 0–4 h after plants are exposed to light (Lee and An 2015). Three technical replicates were included for each sample.

Total RNA was extracted using a Plant RNA extraction kit (Macherey‐Nagel) following the manufacturer's instructions and the principles described by Chang et al. (1993). RNA integrity was assessed by 1.2% agarose gel electrophoresis, confirming the presence of intact 28S and 18S rRNA bands. RNA concentration and purity were measured using an Allsheng Nano‐100 spectrophotometer, ensuring A260/A280 ratios between 1.8 and 2.1.

cDNA was synthesized from 1 μg of purified RNA using the PrimeScript RT reagent kit with a gDNA eraser (Takara Bio Inc.) to remove potential genomic DNA contamination. qRT‐PCR was performed using a Rotor‐Gene TM6000 thermal cycler (Corbett Research) with SYBR Green Real‐Time PCR Master Mix (Toyobo). Gene‐specific primers were designed using Primer3 and validated through a standard dilution series to determine amplification efficiency and specificity. The primers were tested at different concentrations to ensure optimal performance. The qRT‐PCR reaction (total volume of 20 μL) consisted of 10 μL SYBR Green Master Mix, 0.4 μM of each primer, and 1 μL of cDNA template. The cycling conditions were as follows: initial denaturation at 95°C for 3 min, followed by 40 cycles of denaturation at 95°C for 10 s, annealing at 58°C–60°C for 15 s, and extension at 72°C for 20 s. Melt curve analysis was performed to confirm the specificity of the amplification.

Relative gene expression was calculated using the 2^−ΔΔ*Ct*^ method, with *UBIQUITIN* (Ub) as the reference gene (Livak and Schmittgen 2001). Expression values were normalized to a baseline of 1.0, using samples collected 1 day before the onset of day‐length and temperature treatments as the reference. Primer sequences used in this study are listed in Table S1.

### 2.9 Sequence Pretreatment for RNA‐Sequencing

To pretreat the sequenced short reads, duplicated reads produced by PCR during library construction were filtered through in‐house scripts. Final trimmed data were refined using Dynamic‐trim and Length sort of the SolexaQA (Cox et al. 2010) package. Dynamic‐trim renders data as refined cleaned reads by trimming bad quality bases on both ends of short reads according to the Phred score. Length sort eliminates reads with excess base cut off by Dynamic trim. The Phred score was > 20 in Dynamic trim, and the short read length was > 25 bp in Length sort (Figure S3).

### 2.10 Read Mapping

Read mapping was conducted using Bowtie (v.2.1.0) software based on the Langmead and Salzberg (2012) method (mismatch ≤ 2 bp, penalty). Expression was measured by the total number of reads mapped on each gene (Figure S3). The R‐package DEseq‐library (Anders and Huber 2010) was implemented to calculate proper gene expression values for samples with data deviation. To examine the function of selected genes, the annotation information provided by Phytozome (Goodstein et al. 2012) DB was used. The gene expression analysis was conducted using DESeq2, which normalizes raw read counts based on library size and variance estimation. The DEGs were identified using log_2_ (Fold Change) values, with upregulation and downregulation thresholds set at > 1 and < −1, respectively, and an adjusted *p* value (FDR) < 0.01. Normalized counts obtained from DESeq2 were used for downstream statistical analysis and visualization (Anders and Huber 2010).

### 2.11 Selection of Differentially Expressed Genes (DEGs)

To select the differentially expressed genes (DEGs) in each sample, the 2‐fold change (FC) method and the binomial test method (adjusted *p* value (false discovery rate [FDR]) < 0.01) were applied. Upregulated genes were defined as log_2 (FC) > 1, and downregulated as < −1.

### 2.12 Analysis of DEGs Function and Expression Patterns

Alignment was conducted using DEG candidates and sequences provided by Gene ontology (GO)‐Database (DB) for GO analysis (Ashburner et al. 2000). The total number of genes by function was set at counts > 1 for thresholds. The GO depths were set at 3 and categorized into three functional categories: BP (Biological Process), CC (Cellular Component), and MF (Molecular Function). Annotation (filter standard: *e* value ≤ 1^10^, best hits) was conducted with amino acid sequence and BLASTX provided for Kyoto Encyclopedia of Genes and Genomes (KEGG) analysis. Clustering analysis was performed to identify gene expression patterns.

### 2.13 Statistical Analysis

Statistical analyses were performed using R software (version 4.0.3, R Foundation). Significant differences were assessed at *p* < 0.05 using a one‐sample *t*‐test and analysis of variance (ANOVA), followed by Duncan's multiple range test. Prior to statistical analyses, Levene's and Shapiro–Wilk tests were performed to assess the homogeneity of variance and normality of the data. When the assumptions of homogeneity or normality were not met, nonparametric tests, such as the Kruskal–Wallis test, were employed. Hierarchical clustering analysis was conducted using R‐amap (Lucas 2014) and the gplots‐library.

## 3 Results

### 3.1 Temperature and Day Length Interaction Impact on Heading Response

The analysis of days to heading (DTH) under varying temperature and day‐length conditions displayed a consistent pattern in both cultivars. The DTH was shortened in the order of short day‐length and high temperature (SDHT), short day‐length and low temperature (SDLT), long day‐length and high temperature (LDHT), and long day‐length and low temperature (LDLT) (Figure 1A). The average DTH for Odae—temperature‐sensitive cultivar—was 56.4, 61.3, 61.8, and 63.9 days under SDHT, SDLT, LDHT, and LDLT conditions, respectively, and 55.4, 60.1, 65.9, and 67.0 days, respectively, for Saenuri—a photoperiod‐sensitive cultivar—under the same conditions (Figure 1A). The delay in DTH under low temperature (LT) compared to high temperature (HT) was greater under short day‐length (SD) conditions (4.9 days in Odae, 4.7 days in Saenuri) than long day‐length (LD) conditions (2.1 days in Odae, 1.1 days in Saenuri; Figure 1B). Additionally, the DTH delay under LD compared to SD was greater under HT conditions (5.3 days in Odae, 10.4 days in Saenuri) than under LT conditions (2.6 days in Odae, 6.9 days in Saenuri). Furthermore, DTH differences caused by temperature were more substantial in Odae, while day‐length differences were more significant in Saenuri. When averaging the results from both cultivars, DTH was delayed by 4.8 days under LT compared to HT in the SD conditions, while the delay was only 1.6 days in the LD conditions. Similarly, under HT conditions, heading was delayed by 7.9 days in LD compared to SD, and by 4.7 days under LT conditions. Thermosensitivity was 3.0 times higher under SD than LD, indicating a stronger temperature response under SD conditions when averaged across both cultivars. In contrast, photosensitivity was 1.7 times higher under HT than LT, suggesting a stronger response to photoperiod under HT conditions (Figure 1B).

The expression levels of genes promoting flowering (*Hd3a, RFT1, Ehd1*) and repressing flowering (*COL15, Ghd7*) were analyzed during the treatment period. The time series of expression levels at 2–3 days intervals at the same time of day are displayed in Figure S4. The average expression levels over the five time points, representing the overall expression during the treatment period, are shown in Figure 1C. In both cultivars, the expression of the flowering‐promoting genes followed a similar trend to that of the DTH results, increasing in the order of SDHT < SDLT < LDHT < LDLT (Figure 1C). In contrast, the expression of flowering‐repressing genes increased in the order of LDLT, LDHT ≥ SDLT, and SDHT (Figure 1C). Genes promoting flowering showed a clear negative correlation with DTH, whereas genes repressing flowering exhibited a weak positive correlation (Figure 1D).

The three‐way ANOVA results revealed significant impacts of variety, daylength, and temperature on DTH and the expression levels of key flowering‐related genes (Figure 1E). Daylength exhibited the strongest influence on all measured traits, with highly significant *p* values (−log10(*p*) exceeding 8). In addition, temperature had a notable impact, particularly on *DTH, Hd3a*, and *Ghd7* expression. Significant interactions were observed for variety × daylength and daylength × temperature, suggesting that the effects of variety and temperature on gene expression were dependent on photoperiod conditions (Figure 1E). In contrast, the three‐way interaction (variety × daylength × temperature) was not statistically significant for most genes, indicating that the combined effects of these factors were largely additive rather than synergistic (Figure 1E). These results indicate that the effect of daylength on heading response is more pronounced under HT conditions, whereas the effect of temperature on heading response is more pronounced under short‐day conditions (Figure 1E).

### 3.2 Reduced Expression of Photosynthesis Genes Under Heading‐Promoting Conditions

To comprehensively assess transcriptional responses to photoperiod and temperature, RNA‐Seq analysis was conducted across three tissue types (leaf, lower stem, and stem vs. leaf). Differentially expressed genes (DEGs) were identified and subjected to GO enrichment and hierarchical clustering analysis (Figure S5; Tables [Link], [Link]). KEGG pathway enrichment based on these DEGs revealed distinct transcriptional shifts in response to environmental conditions.

**FIGURE 2:**
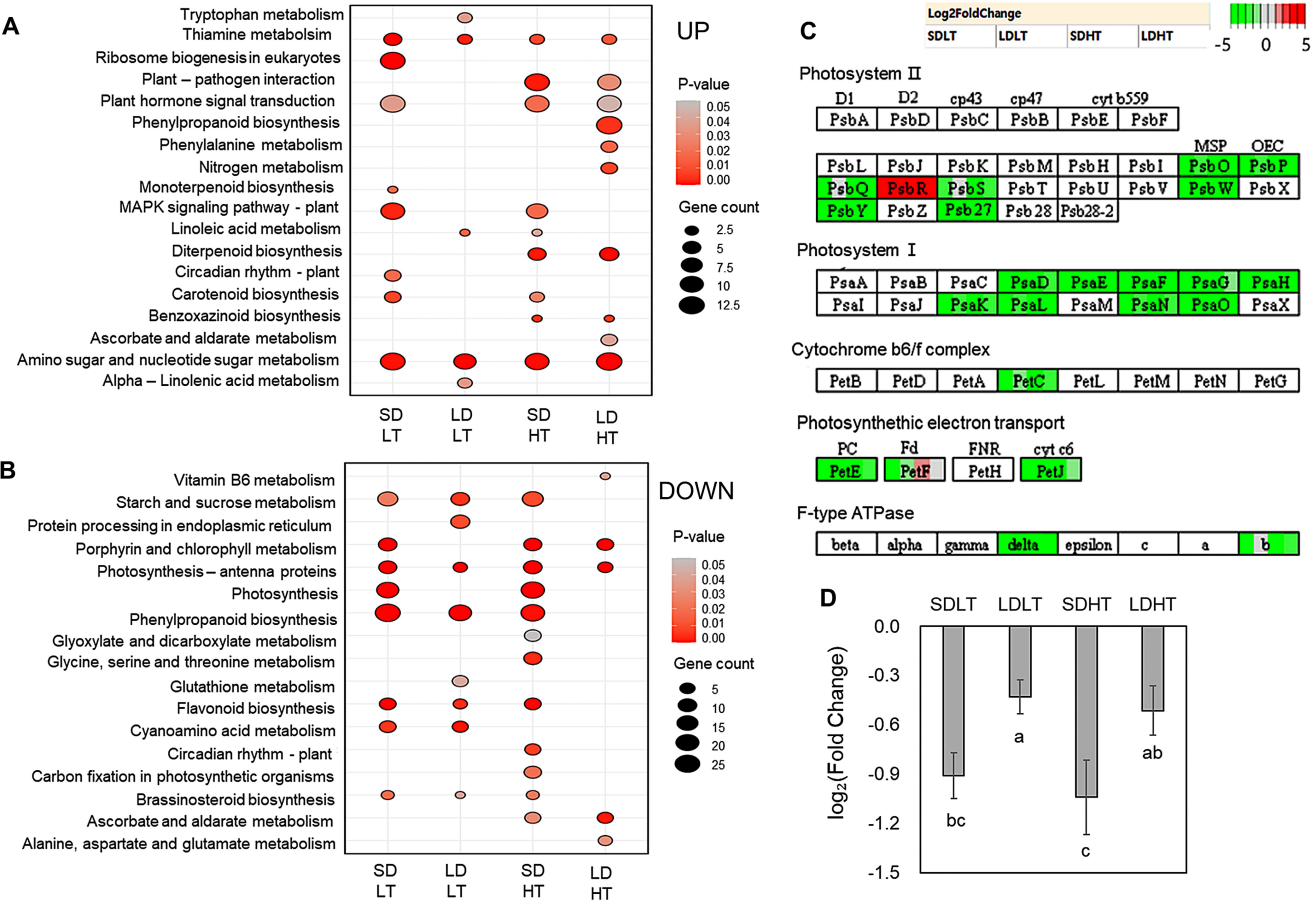
KEGG pathway enrichment analysis and expression patterns of differentially expressed genes (DEGs) under different day‐length and temperature conditions in leaf. (A) The KEGG pathway enrichment analysis of upregulated DEGs (*p* < 0.05). (B) KEGG pathway enrichment analysis of downregulated DEGs (*p* < 0.05). (C) Expression patterns of photosynthesis‐related genes mapped onto KEGG pathways. (D) Log_2_ (fold change) values of photosynthesis‐related genes under different conditions. Different letters indicate significant differences within each dataset based on Duncan's multiple range test (*p* < 0.05). The RNA‐Seq data were obtained from three biological replicates (*n* = 3), collected at two time points (1 day before treatment and 2 days after treatment) for each condition. Gene expression levels 2 days after treatment were compared to those at 1 day before treatment within each temperature and day‐length condition.

The KEGG pathway analysis of upregulated DEGs under each treatment condition revealed that pathways simultaneously responding to day‐length treatments (SDLT and SDHT) included the MAPK signaling pathway–plant and carotenoid biosynthesis (Figure 2A; Table S8). Pathways simultaneously responding to temperature treatments (SDHT and LDHT) included diterpenoid biosynthesis (Figure 2A). Pathways responding under all treatment conditions included thiamine metabolism and amino sugar and nucleotide sugar metabolism.

Conversely, KEGG pathway analysis of downregulated DEGs under each treatment condition revealed that pathways simultaneously responding to day‐length treatments (SDLT and SDHT) included the photosynthesis pathway (Figure 2B; Table S8). Pathways simultaneously responding to temperature treatments included ascorbate and aldarate metabolism (SDLT and LDLT) in addition to cyanoamino acid metabolism (SDHT and LDHT) (Figure 2B). Photosynthesis–antenna proteins were identified as the pathway responding under all treatment conditions.

The expression patterns of genes associated with photosynthesis‐related pathways (Photosystem I, Photosystem II, Cytochrome b6/f complex, photosynthetic electron transport, and F‐type ATPase) were further investigated (Figure 2C; Table S9). Most genes, excluding *PsbR*, were downregulated compared to pre‐treatment conditions (Figure 2C; Table S9). Notably, the average expression levels of photosynthesis‐related genes followed a decreasing trend (Figure 2D), which was consistent with the pattern of shorter DTH in the order of SDHT, SDLT, LDHT, and LDLT (Figure 1A). This trend was opposite to the expression patterns of heading‐promoting genes (Figure 1B).

### 3.3 Glyceric Acid as a Key Metabolite Associated With Heading Response

**FIGURE 3:**
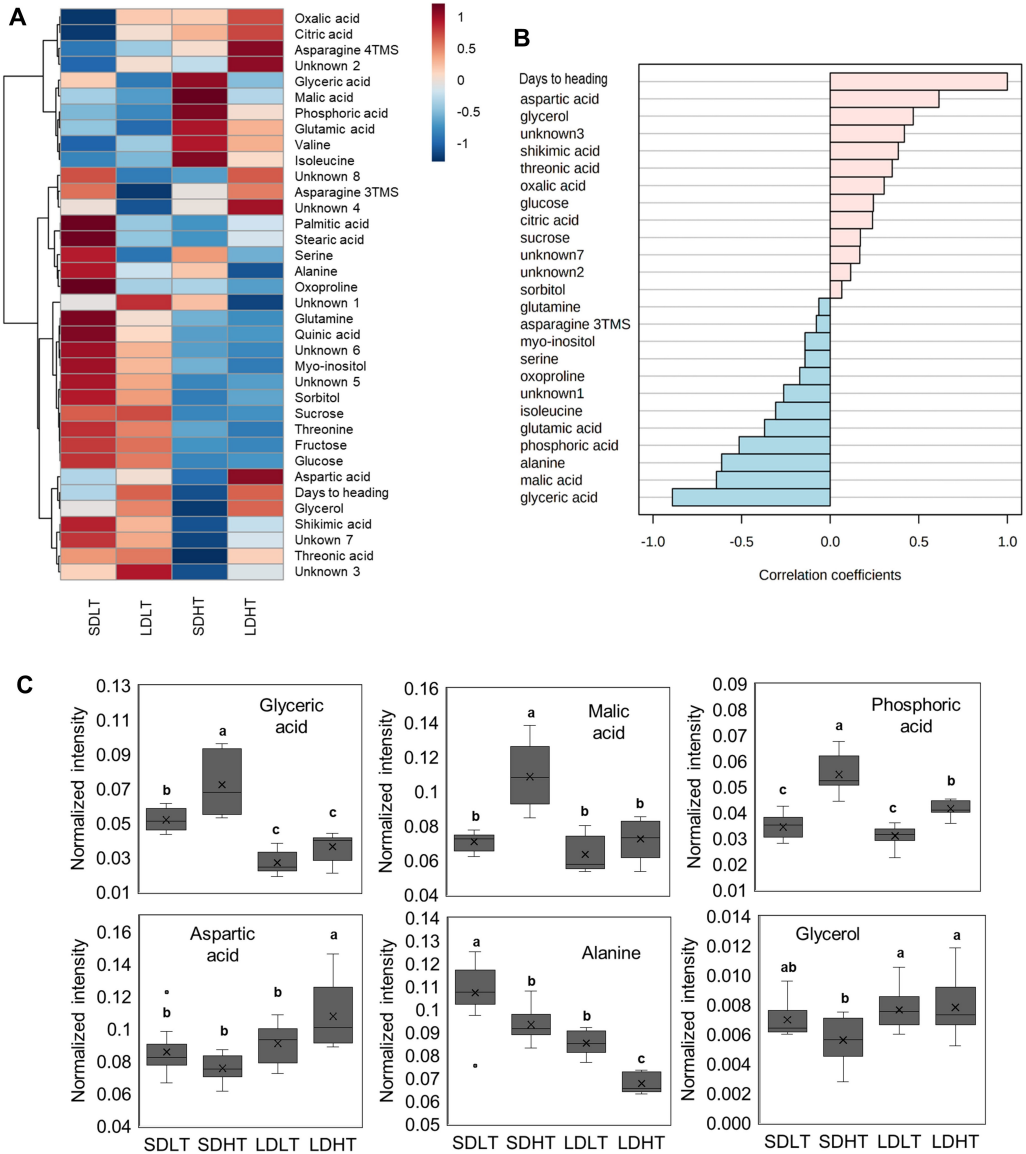
Metabolomic analysis of rice leaves under temperature and photoperiod treatments. (A) Hierarchical clustering heatmap of metabolite profiles under different temperature and photoperiod conditions. *Z*‐score normalization was applied to chromatogram intensity values for visualization (MetaboAnalyst 5.0). (B) Correlation coefficients of individual metabolites with days to heading, ranked in descending order. (C) Box plots of the six metabolites with the strongest absolute correlation coefficients to days to heading. The black horizontal lines indicate the median, and the “×” symbol represents the mean. Different letters (a–c) indicate significant differences within each metabolite based on Duncan's multiple range test (*p* < 0.05, *n* = 9 per treatment).

Metabolite profile hierarchical clustering analysis revealed distinct patterns of metabolite concentration changes in response to temperature (LT, HT) and daylength (SD, LD) treatments (Figures 3A, S6, and S7). In total, 35 metabolites were analyzed, with 14 metabolites, including glutamine, sucrose, and fructose, exhibiting higher concentrations under LT conditions and lower concentrations under HT conditions. In contrast, six metabolites, including phosphoric acid, glutamic acid, and valine, displayed an inverse trend, with increased concentrations under HT conditions and reduced levels under LT conditions. Meanwhile, three metabolites (aspartic acid, glycerol, and Unknown 2) displayed higher concentrations under LD conditions and lower concentrations under SD conditions, while glyceric acid and malic acid exhibited the opposite trend. Overall, 20 metabolites were temperature‐responsive, 5 were primarily influenced by daylength, and 10 displayed ambiguous patterns.

Among the top 25 metabolites, 12 exhibited positive correlations with DTH, while 12 displayed negative correlations (Figure 3B). Notably, glyceric acid exhibited the strongest negative correlation (*r* = −0.89184, *p* < 0.0001), whereas aspartic acid displayed the highest positive correlation (*r* = 0.6136, *p* < 0.001).

The top six metabolites with the highest absolute correlation coefficients were further analyzed using box plots (Figure 3C). Among them, glyceric acid, malic acid, phosphoric acid, and alanine negatively correlated with DTH, while aspartic acid and glycerol exhibited positive correlations. Among the negatively correlated metabolites, glyceric acid, malic acid, and phosphoric acid exhibited similar patterns across treatments. Glyceric acid, with the strongest negative correlation, displayed normalized intensity values of 0.073 (SDHT), 0.052 (SDLT), 0.037 (LDHT), and 0.027 (LDLT), with its accumulation following the order of shorter heading time: SDHT > SDLT > LDHT > LDLT. This finding suggests that glyceric acid accumulation is inversely associated with heading time.

### 3.4 Photoperiod‐ and Temperature‐Dependent Regulation of Glycerate Metabolism

**FIGURE 4:**
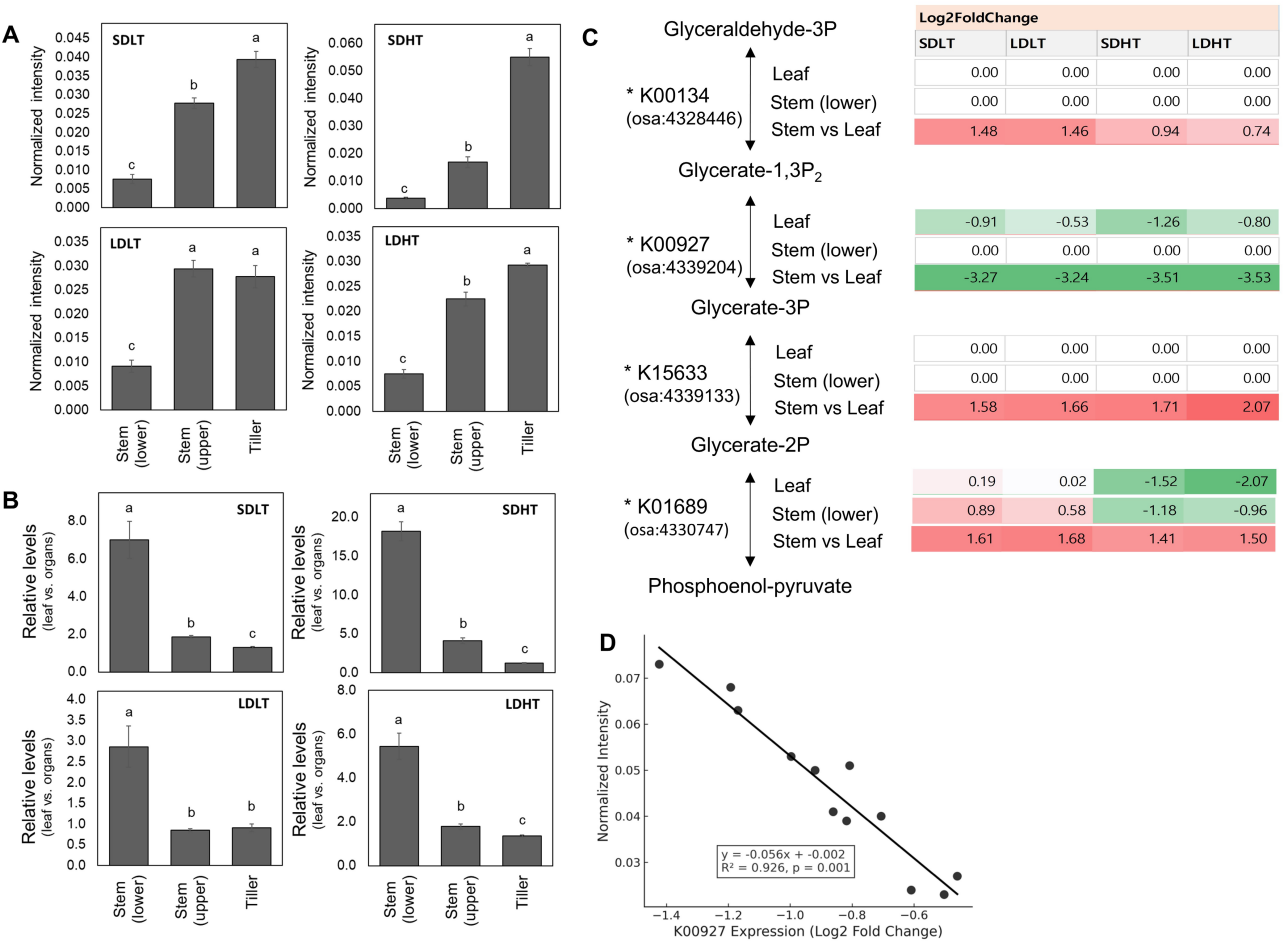
Effects of temperature, daylength treatments, and organ specificity on glycerate concentration (normalized chromatogram intensity) and gene expression involved in glycolysis metabolism (KEGG pathway). (A) Glycerate concentration in different organs, including the lower stem, upper stem, and tillers. (B) Relative glycerate concentration in each organ compared to the leaf. (C) Changes in the relative expression (Log_2_ fold change) of genes involved in glycolysis metabolism in the leaf, lower stem, and lower stem relative to the leaf, from glyceraldehyde‐3‐phosphate to phosphoenolpyruvate. (D) Correlation between glycerate content and K00927 expression. Different letters (a–c) indicate significant differences within each metabolite based on Duncan's multiple range test (*p* < 0.05, *n* = 9 per treatment). Sampling locations for each organ are shown in Figure S2.

Glycerate is an intermediate in the photorespiration and photosynthesis processes, used in the form of 3‐PGA in the glycolysis Calvin cycle, and is required for serine synthesis in the glycerate‐serine pathway (Igamberdiev and Kleczkowski 2018; Kleczkowski and Igamberdiev 2024). To investigate the effects of temperature and day length on glycerate metabolism in rice, glycerate concentrations and the expression of glycolysis‐related genes in different plant organs were analyzed, including the main stem (lower and upper segments) and tillers (tiller stems and leaves), under various temperature and photoperiod conditions (Figure 4).

Glycerate concentrations exhibited significant variation among the organs, excluding leaves, in response to temperature and daylength treatments (Figure 4A). Under SDLT conditions, glycerate levels were significantly higher in the tiller (0.039 ± 0.002) than in the upper (0.028 ± 0.001) and lower (0.008 ± 0.001) stem (*p* < 0.05). A similar trend was observed under SDHT conditions, with the tiller exhibiting the highest glycerate concentration (0.055 ± 0.003), followed by the upper (0.017 ± 0.002) and lower (0.004 ± 0.001) stem.

In the tiller, the glycerate levels were higher under short‐day conditions than under long‐day conditions. In contrast, the upper and lower stem displayed relatively small differences across treatments (Figure 4A). Notably, in the tiller, a significant temperature‐dependent difference was observed under short‐day conditions (0.055 ± 0.003 in SDHT vs. 0.039 ± 0.002 in SDLT), whereas glycerate concentrations remained constant regardless of temperature under long‐day conditions (0.028 ± 0.002 in LDLT and 0.029 ± 0.001 in LDHT). This trend aligns with the variation in heading time observed under different photoperiod conditions, suggesting a potential link between glycerate metabolism and photoperiod‐dependent developmental responses.

The relative glycerate concentration in the leaf compared to the stem and tiller exhibited a strong dependence on temperature and daylength conditions (Figure 4B). Glycerate accumulation in the leaf was approximately 7.0 times higher under SDLT conditions than in the lower stem, whereas the upper stem and tiller displayed significantly lower relative values compared to the leaf (1.9 and 1.3, respectively). A similar pattern was observed under SDHT conditions, where the relative glycerate content in the leaf was 18.2 times higher than in the lower stem, while lower ratios were observed in the upper stem (4.1) and tiller (1.2).

Under LDLT and LDHT conditions, the relative glycerate concentration in the leaf remained higher than in the other organs; however, the difference was less pronounced than under SD conditions. In LDLT, the leaf had 2.9 times higher glycerate levels than the lower stem, while the upper stem and tiller exhibited markedly lower relative values (0.9 and 0.9, respectively). Similarly, under LDHT, the relative glycerate concentration in the leaf was 5.4 times higher than in the lower stem, while the upper stem (1.8) and tiller (1.4) displayed smaller differences (Figure 4B). These results indicate that glycerate accumulation in the leaf was significantly higher than in other organs across all treatments, with the largest differences under SDHT conditions. Notably, the disparity between the leaf and the lower stem was more pronounced under short‐day conditions, particularly at high temperatures.

To investigate the molecular regulation of glycerate metabolism, RNA‐Seq‐based KEGG pathway analysis was conducted to examine the expression of key glycolysis‐related genes in the leaf, lower main stem, and lower main stem relative to the leaf. The expression levels of key enzymes involved in glycolysis, particularly those catalyzing reactions from glyceraldehyde‐3‐phosphate to phosphoenolpyruvate, exhibited organ‐specific and treatment‐dependent variations (Figure 4C; Table S10). In leaves, phosphoglycerate kinase (K00927) was downregulated under all conditions, with the strongest suppression under SDHT (−1.26), followed by SDLT (−0.91), LDHT (−0.80), and LDLT (−0.53). This indicated that its expression was primarily influenced by photoperiod. In contrast, 2‐phospho‐D‐glycerate hydro‐lyase (K01689) exhibited a distinct response pattern, showing stronger downregulation in response to high temperature than photoperiod. Its suppression was most pronounced under LDHT (−2.07) and SDHT (−1.52), while remaining relatively stable under SDLT (0.19) and LDLT (0.02). These results suggest that phosphoglycerate kinase (K00927) is primarily regulated by photoperiod, whereas 2‐phospho‐D‐glycerate hydro‐lyase (K01689) is more sensitive to temperature. In contrast, D‐phosphoglycerate 2,3‐phosphomutase (K15633) remained unchanged across all treatments, indicating that its expression was less influenced by environmental factors.

In the lower stem, the expression of 2‐phospho‐D‐glycerate hydro‐lyase (K01689) exhibited an opposite trend, showing upregulation under SDLT (0.89) and LDLT (0.58) and downregulation under SDHT (−1.18) and LDHT (−0.96), reinforcing its temperature‐dependent regulation (Figure 4C). A comparative analysis between the lower stem and leaf revealed that D‐phosphoglycerate 2,3‐phosphomutase (K15633) (1.58–2.07) and 2‐phospho‐D‐glycerate hydro‐lyase (K01689) (1.41–1.68) were upregulated in the lower stem relative to the leaf, whereas phosphoglycerate kinase (K00927) remained consistently downregulated (−3.24 to −3.53). In leaves, the strongest suppression of K00927 was under short‐day conditions, particularly SDHT, coinciding with the highest glycerate accumulation in the leaf (Figure 3C). The linear regression analysis confirmed a significant negative correlation between K00927 expression and glycerate concentration (*R* ^2^ = 0.926, *p* < 0.001, Figure 4D), indicating that reduced K00927 expression is strongly associated with increased glycerate accumulation.

Glyceric acid exhibited the strongest negative correlation with DTH (*r* = −0.89184, *p* < 0.0001) among the top‐ranked metabolites. This suggests that glycerate metabolism may be functionally linked to photoperiod‐dependent heading regulation; given its strong association with K00927 expression, K00927 may be critical in metabolic pathways involved in heading control.

### 3.5 Organ‐Specific Regulation of Serine Metabolism in Response to Temperature and Photoperiod

**FIGURE 5:**
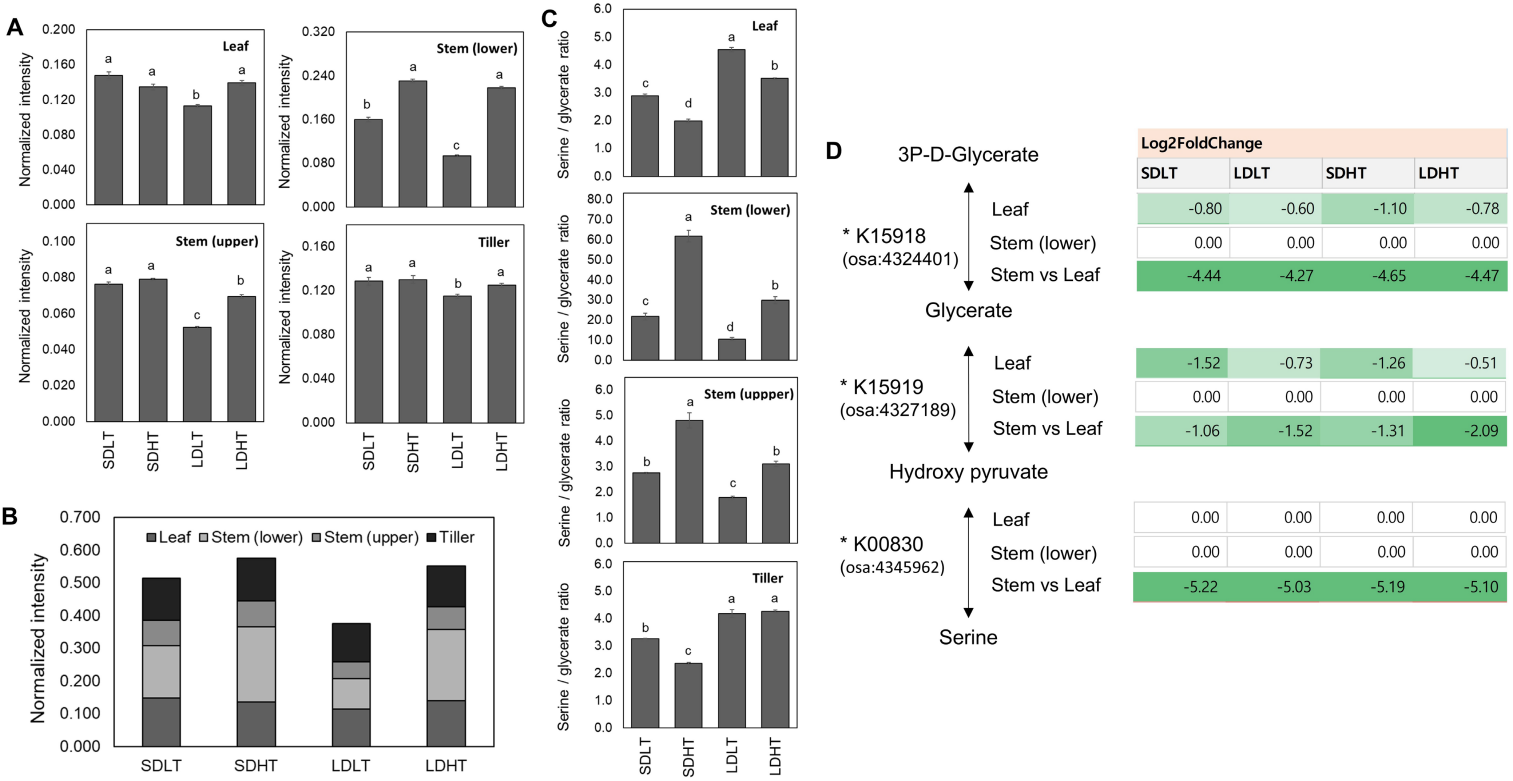
Effects of temperature, daylength treatments, and organ specificity on serine concentration (normalized chromatogram intensity) and the expression of genes involved in glycine, serine, and threonine metabolism (KEGG pathway). (A) Serine concentration in different organs, including the leaf, lower stem, upper stem, and tillers. (B) Stacked bar graph representation of serine concentrations across organs under each treatment condition. (C) Changes in the serine‐to‐glycerate ratio across different organs and treatment conditions. (D) Changes in the relative expression (Log_2_ fold change) of genes involved in glycine, serine, and threonine metabolism in the leaf, lower stem, and tillers relative to the leaf, from 3P‐D‐glycerate to serine. Different letters (a–c) indicate significant differences within each metabolite based on Duncan's multiple range test (*p* < 0.05, *n* = 9 per treatment). Figure S2 displays the sampling locations for each organ.

Serine concentrations varied significantly depending on the treatment conditions and organ type (Figure 5A). Serine levels in the leaf and tiller remained relatively stable across treatments. However, in stem tissues, serine concentrations were significantly higher under short‐day conditions when temperature remained constant. In the lower stem, serine accumulation was highest under SDHT (0.231) and LDHT (0.218), whereas the lowest concentration was observed under LDLT (0.094), indicating substantial variation across treatments. Similarly, in the upper stem, serine levels were significantly higher under SDLT (0.076) and SDHT (0.079), whereas LDLT (0.052) exhibited the lowest concentration. Thus, under the same temperature conditions, serine accumulation is higher under short‐day conditions in both upper and lower stem tissues.

Across different organs, serine concentrations exhibited significant variation (Figure 5A). The lower stem had the highest serine concentration (average: 0.176), while the upper stem had the lowest (0.069), with intermediate levels in the leaf (0.134) and tiller (0.125). Temperature and photoperiod effects were more pronounced in the stem than in the leaf and tiller. The total serine concentrations across treatments (Figure 5B) further highlight the organ‐specific distribution of serine metabolism. The lower stem exhibited the highest total serine accumulation (0.703), followed by the leaf (0.535) and tiller (0.499), with the upper stem showing the lowest total concentration (0.277). This pattern is consistent with the organ‐specific averages, reinforcing that serine accumulation is highest in the lower stem and lowest in the upper stem, while the leaf and tiller maintain intermediate levels.

The serine‐to‐glycerate ratio—an indicator of serine metabolism efficiency—exhibited significant organ specificity and treatment‐dependent variation (Figure 5C). Among all organs, the lower stem displayed the highest ratios and the greatest variation, ranging from 10.6 (LDLT) to 61.7 (SDHT). This suggests that serine metabolism in the lower stem is highly dynamic and strongly influenced by environmental conditions. Notably, the ratio was significantly higher under SDHT (61.7) and LDHT (29.8), while LDLT (10.6) exhibited the lowest ratio. In contrast, the upper stem exhibited markedly lower serine‐to‐glycerate ratios than the lower stem, ranging from 1.8 (LDLT) to 4.8 (SDHT). This indicates that serine metabolism in the upper stem is more restricted compared to the lower stem. The leaf and tiller showed relatively stable serine‐to‐glycerate ratios across treatments, with ratios ranging from 2.0 (SDHT) to 4.5 (LDLT) in the leaf and 2.4 (SDHT) to 4.3 (LDHT) in the tiller. Hence, the leaf and tiller serine metabolism is less sensitive to temperature and photoperiod fluctuations than stem tissues. Overall, the lower stem was identified as the primary site of serine metabolism regulation, which exhibited both the highest serine concentrations and the most pronounced fluctuations in the serine‐to‐glycerate ratio across treatments.

To examine the regulatory mechanisms underlying serine metabolism, RNA‐Seq‐based KEGG pathway analysis was conducted to analyze the expression of genes involved in glycine, serine, and threonine metabolism (Figure 5D; Table S11). In the leaf, D‐glycerate 3‐kinase (K15918) was consistently downregulated across all treatments, with the strongest suppression under SDHT (−1.10). In contrast, glyoxylate/hydroxypyruvate reductase (K15919) showed the greatest downregulation under SDLT (−1.52), instead of SDHT (−1.26). These results suggest that different environmental conditions selectively influence the expression of key enzymes in serine metabolism. However, serine‐pyruvate transaminase (K00830) expression remained unchanged across all treatments, indicating that upstream steps of serine biosynthesis were more influenced by environmental factors than transamination processes.

A comparative analysis of stem and leaf expression 2 days after treatment revealed that D‐glycerate 3‐kinase (K15918) was strongly downregulated in the lower stem compared to the leaf (−4.44 to −4.55 across treatments), while glyoxylate/hydroxypyruvate reductase (K15919) displayed moderate downregulation (−1.06 to −2.09), and serine‐pyruvate transaminase (K00830) was highly suppressed (−5.03 to −5.22). Despite the higher serine accumulation in the lower stem, these results indicate that serine biosynthesis is transcriptionally suppressed in the lower stem relative to the leaf, suggesting that serine accumulation in the lower stem is predominantly regulated through non‐transcriptional mechanisms.

### 3.6 Effects of Glycerate Treatment on Heading Across Different Concentrations and Temperature

**FIGURE 6:**
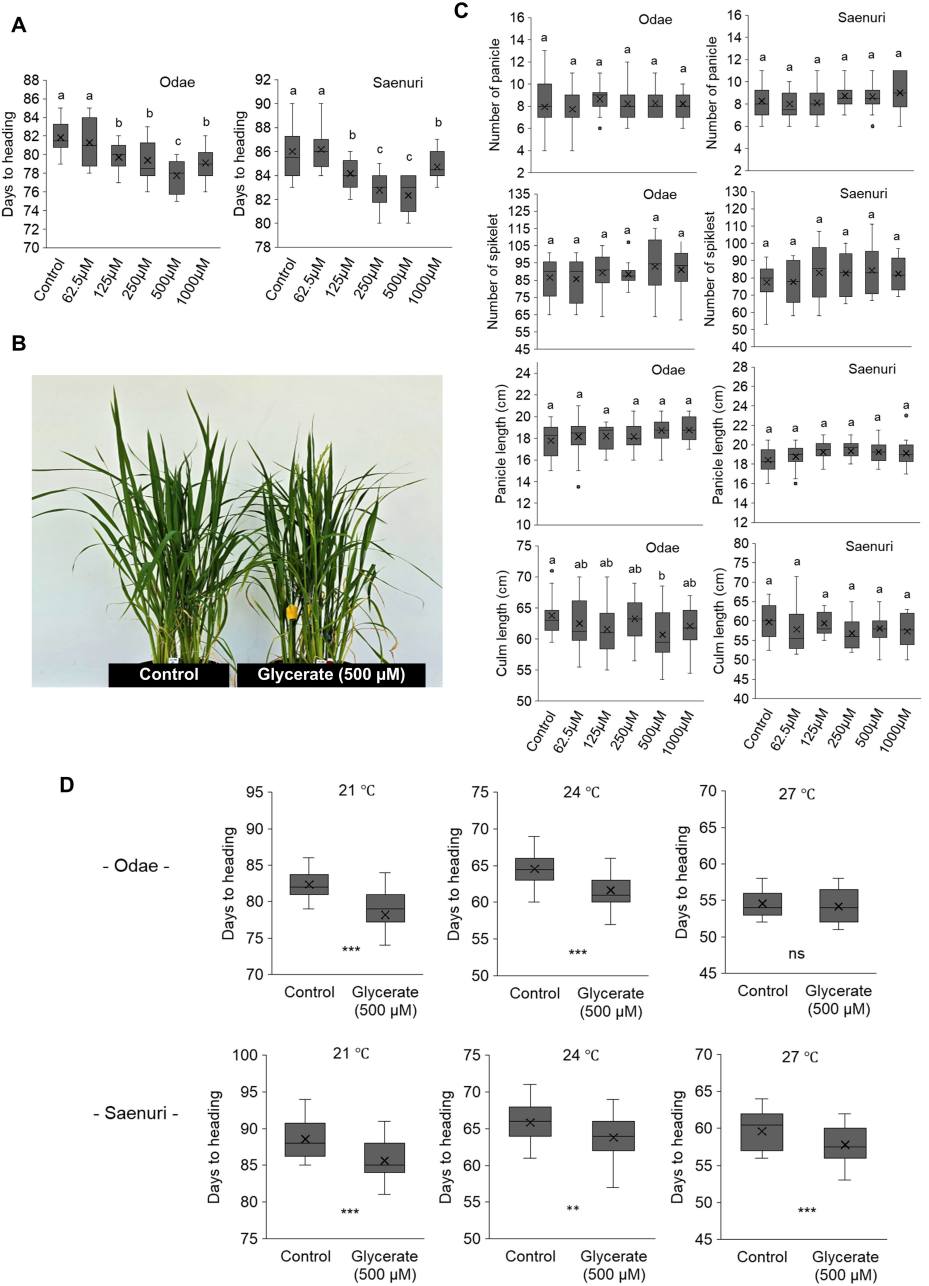
Effects of glycerate treatment on heading date and plant morphology in Odae and Saenuri rice cultivars. (A) Box plots displaying DTH under different glycerate concentrations (62.5, 125, 250, 500, and 1000 μM) compared to that of the control (*n* = 18 per group). (B) Representative images of Saenuri plants under control and 500 μM glycerate treatment, illustrating differences in growth and heading. (C) Box plots showing the effects of glycerate treatment on growth parameters at the heading stage under different temperatures (*n* = 18 per group). The black horizontal lines indicate the median, and the “×” symbol represents the mean. Panicle number was measured per hill, and spikelet number was measured per panicle. (D) Box plots illustrating the effects of glycerate treatment on heading date under different temperature conditions. A two‐sample t‐test was used to compare the Control and Glycerate groups (*n* = 36 per group). Statistical significance levels: ***p < 0.001, **p < 0.01, *p < 0.05, ns = not significant. Different letters (a–c) indicate significant differences within each group based on Duncan's multiple range test (*p* < 0.05).

Glycerate application significantly influenced heading date in Odae and Saenuri rice cultivars (Figure 6A). In Odae, heading was accelerated at ≥ 125 μM, with the shortest DTH observed at 500 μM (77.8 days, *p* < 0.05) compared to the control (81.8 days). Similarly, in Saenuri, heading was significantly promoted under 250 and 500 μM glycerate treatments, with DTH reduced to 82.8 and 82.3 days, respectively (*p* < 0.05) compared to the control (86.0 days). However, at the highest glycerate concentration (1000 μM), heading time was slightly delayed in both cultivars compared to 250–500 μM treatments, though it remained shorter than the control.

Representative images of Saenuri plants under control and 500 μM glycerate treatments illustrate visible differences in plant growth and heading stage (Figure 6B). Glycerate treatment did not impact most growth parameters, including panicle number, spikelet number, and panicle length, in either cultivar (Figure 6C). However, a slight increasing trend in spikelet number was observed at 500 and 1000 μM in Odae. In Odae, culm length was significantly reduced at 500 μM glycerate treatment (*p* < 0.05) compared to the control, while no significant reduction was observed in Saenuri. Despite the lack of statistically significant differences in most growth traits, these results suggest that glycerate may have a minor effect on spikelet development and culm elongation, particularly in Odae.

To evaluate the effect of glycerate treatment across different growth durations, temperature conditions were adjusted to create varying growth periods, and 500 μM glycerate, the most effective concentration, was applied within each temperature treatment (Figure 6D). Across temperature conditions, glycerate consistently accelerated heading in both cultivars, with a more pronounced effect at lower temperatures. At 21°C, heading was significantly earlier in both cultivars (Odae: 82.4 → 78.2 days, *p* < 0.001; Saenuri: 88.6 → 85.6 days, *p* < 0.001). A similar trend was observed at 24°C, where heading time was reduced in Odae (64.6 → 61.6 days, *p* < 0.001) and Saenuri (65.4 → 63.8 days, *p* < 0.05). At 27°C, heading time occurred slightly earlier in glycerate‐treated plants; although this effect was statistically significant in Saenuri (*p* = 0.0033), it was insignificant in Odae (*p* = 0.4142). These results indicate that the heading‐promoting effect of glycerate is strongest at moderate temperatures (21°C–24°C), under longer growth durations, and becomes less effective at higher temperatures (27°C), with shortened growth periods. This suggests that the impact of glycerate treatment is more pronounced when the overall growth duration is extended—particularly in Odae.

## 4 Discussion

### 4.1 Photoperiod and Temperature Interactions in Heading Regulation

Our results indicate that the effect of temperature on heading response is more pronounced under short‐day conditions, whereas photoperiod effects are stronger under high‐temperature conditions (Figure 1B). Hence, temperature sensitivity is enhanced under short‐day conditions, while photoperiod plays a dominant role in heading regulation (Figure 1). Accordingly, the interaction between photoperiod and temperature is not merely additive and may involve a regulatory mechanism in which short‐day conditions amplify the effect of temperature on heading response (Figure 1C,E).

Consistent with this, previous studies have suggested that photoperiod and temperature signals interact to regulate heading in rice, rather than acting independently. For example, Luan et al. (2009) reported that low temperatures suppress *Hd3a* expression regardless of photoperiod, while photoperiodic responses can still modulate heading under such conditions via potential post‐translational regulation of *Hd1*. Additionally, Song and Luan (2012) highlighted that photoperiod and temperature are integrated through interconnected pathways, including short‐day promotion, long‐day suppression, and temperature‐mediated regulation of flowering genes. These findings collectively support the hypothesis that photoperiod and temperature cues are integrated within complex regulatory networks to control floral induction in rice, reinforcing our interpretation of their dynamic crosstalk in heading regulation.

### 4.2 Downregulation of Photosynthesis Under Heading‐Promoting Conditions

RNA‐Seq analysis was conducted under well‐defined temperature and photoperiod conditions, revealing clear differences in heading‐related gene expression across treatments (Figure 1C). The KEGG pathway analysis revealed that photosynthesis‐related pathways were significantly downregulated under conditions that strongly promote heading, such as SDHT (Figure 2B). Further examination of individual gene expression patterns showed that most photosynthesis‐related genes, including those involved in photosystem I, photosystem II, cytochrome b6/f complex, and ATP synthesis, were downregulated under SDHT (Figure 2C,D). This trend strongly correlated with heading acceleration, exhibiting an opposite pattern to the expression of flowering‐promoting genes (Figure 1C).

Although previous studies have shown that high light intensity promotes flowering in long‐day plants such as Arabidopsis, similar studies on rice remain limited (Cho et al. 2024; Lee et al. 2023). As a short‐day plant, rice may exhibit a different regulatory response to light conditions than Arabidopsis. In *Arabidopsis*, sucrose and trehalose‐6‐phosphate (*T6P*) have been identified as pivotal metabolic signals promoting flowering (Cho et al. 2018), while in rice, sucrose has been shown to facilitate *Ghd7* degradation via K48‐linked polyubiquitination, thereby accelerating heading (Cho et al. 2024). Moreover, recent studies have suggested solar radiation levels can influence rice heading similarly to temperature, highlighting the importance of light and energy availability in floral induction (Kim et al. 2025). Short‐day conditions may reduce the total daily light exposure, thereby lowering photosynthetic activity. This could lead to a metabolic shift from vegetative to reproductive growth, a phenomenon also observed under low nitrogen conditions, where flowering is promoted as a response to reduced vegetative growth capacity (Ye et al. 2019). However, this hypothesis remains to be tested.

### 4.3 Glycerate Accumulation and Its Link to Heading Regulation

Considering the observed photosynthesis suppression under heading‐promoting conditions, metabolites associated with carbon metabolism and photorespiration were hypothesized to help regulate heading time. Metabolomic analysis revealed that glycerate, a key metabolite in the photorespiration pathway (Igamberdiev and Kleczkowski 2018; Kleczkowski and Igamberdiev 2024), exhibited the strongest correlation with heading response (Figure 3B). The negative correlation between glycerate accumulation and DTH suggests that higher glycerate levels may be associated with earlier heading.

Further analysis showed that glycerate concentrations were significantly higher under SDHT and SDLT compared to LDHT and LDLT, aligning with the observed acceleration of heading (Figures 3C and 4A). This pattern was also evident in relative organ‐specific glycerate accumulation, where glycerate levels were highest in the leaf under heading‐promoting conditions (Figure 4B). To improve our understanding of glycerate metabolic regulation, the expression of glycolysis‐related genes was examined in different organs under varying temperature and photoperiod conditions (Figure 4C). A key finding was the strong downregulation of phosphoglycerate kinase (PGK, K00927) under short‐day conditions, particularly under SDHT, which coincided with increased glycerate accumulation in the leaf (Figure 4C,D). Considering that PGK is responsible for converting 1,3‐bisphosphoglycerate to 3‐phosphoglycerate (3‐PGA) in glycolysis, its suppression could lead to reduced glycolytic flux and rerouting of metabolic intermediates toward glycerate accumulation (Rosa‐Téllez et al. 2018). However, further metabolic flux analysis is required to confirm whether this enzymatic regulation directly influences glycerate levels.

A previous study using a rice glycolate/glycerate translocator mutant found that high glycerate accumulation was associated with reduced photosynthetic efficiency, leading to lower chlorophyll content and a decrease in tiller numbers (Shim et al. 2020). Although this study did not investigate heading regulation directly, it suggests that glycerate accumulation may influence plant growth and developmental transitions by altering photosynthetic metabolism. In this study, higher leaf glycerate content was associated with earlier heading, shorter culm length, and a slight increase in spikelet number (Figure 6A,C). This aligns with the hypothesis that glycerate may be linked to the shift from vegetative to reproductive growth, potentially influencing the timing of heading.

### 4.4 Organ‐Specific Regulation of Serine Metabolism and Its Relationship to Glycerate

In addition to glycerate, serine metabolism was analyzed due to its close biochemical link to glycerate through the glycerate‐serine pathway (Igamberdiev and Kleczkowski 2018; Zelcbuch et al. 2015). In contrast to glycerate, serine levels remained relatively stable in the leaf and tiller but exhibited significant variation in stem tissues, particularly in the lower stem (Figure 5A,B). The serine‐to‐glycerate ratio was highest in lower stem tissues under SDHT, suggesting enhanced serine biosynthesis in response to environmental conditions (Figure 5C). Strikingly, the serine‐to‐glycerate ratio in the lower stem was more than 10 times higher than in the upper stem, a difference not observed in other organs (Figure 5C). This suggests that serine metabolism is regulated differentially within stem tissues, potentially playing a distinct role in floral induction processes.

At the transcriptional level, serine biosynthesis genes exhibited minimal changes in the lower stem despite high serine accumulation, indicating that serine may be translocated from other organs instead of being synthesized locally (Figure 5D). This hypothesis is supported by previous reports demonstrating that serine is a mobile amino acid transported using the phloem in multiple plant species (Liu et al. 2024; Ros et al. 2014).

The cause of the considerable difference in serine levels between the lower and upper stem remains unclear; however, it may be related to the spatial localization of the shoot apical meristem, where floral induction occurs, as it is typically positioned in the lower stem region (Chongloi et al. 2019); hence, the metabolic changes observed in this tissue may be directly relevant to heading regulation (Figure 6).

### 4.5 Physiological Effects of Glycerate Treatment on Heading and Growth

To further investigate the functional role of glycerate in heading regulation, exogenous glycerate was applied at different concentrations and its effects on heading time and growth analyzed (Figure 6). Glycerate treatment exhibited a concentration‐dependent response, with the most pronounced heading acceleration observed at 250–500 μM, while at 1000 μM, the effect was diminished (Figure 6A) This suggests that glycerate has an optimal concentration range for promoting heading, beyond which its effect is reduced, possibly due to metabolic feedback inhibition. Despite its clear impact on heading time, glycerate treatment had minimal effects on overall plant growth parameters. While culm length was slightly reduced at 500 μM in Odae, and spikelet number displayed a slight increasing trend, these changes were not significant (Figure 6C).

Temperature‐dependent analysis further revealed that glycerate's heading‐promoting effect was strongest at 21°C–24°C; however, it diminished at 27°C (Figure 6D). To understand this, we considered two factors. First, at 27°C, the DTH was notably shortened, indicating an overall reduction in the growth period. Second, as described in the Section 2, our study applied glycerate treatments uniformly across all temperature conditions from 10 to 31 days after transplantation. However, higher temperatures accelerated the developmental transition from the PSP to the PPP, meaning that, despite the same treatment duration, the actual number of days during which glycerate was applied within the PSP period was reduced at higher temperatures. These combined factors likely contributed to the reduced effectiveness of glycerate treatment at 27°C.

### 4.6 Implications and Future Research

This study demonstrates that metabolic adjustments, in response to photoperiod and temperature, play a crucial role in rice heading regulation. Glycerate and serine metabolism were identified as key metabolic pathways associated with heading responses, highlighting their potential involvement in floral induction. Specifically, the effects of temperature on heading are more pronounced under short‐day conditions, suggesting a regulatory interaction between photoperiod and temperature in floral transition. Additionally, photosynthesis‐related pathways are significantly downregulated under heading‐promoting conditions, indicating a possible metabolic shift that facilitates reproductive development.

**FIGURE 7:**
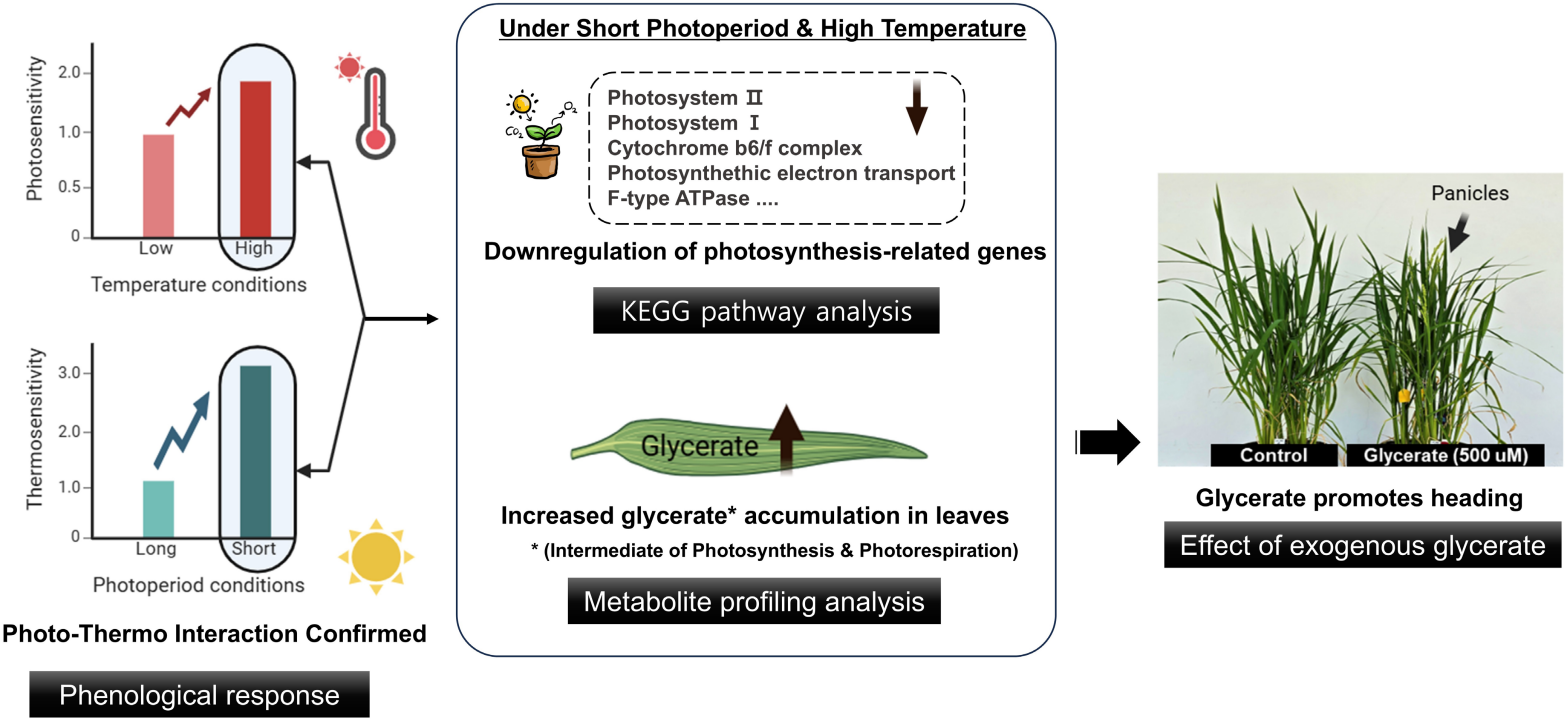
Conceptual summary of photoperiod–temperature interaction and glycerate‐associated heading response in rice.

Glycerate accumulation strongly correlated with heading acceleration, with organ‐specific distribution patterns suggesting its potential role as a metabolic signal for floral induction. Serine metabolism also displayed distinct organ‐specific regulation, particularly in the lower stem, where floral transition occurs. Furthermore, exogenous glycerate application accelerated heading in a concentration‐dependent manner, with temperature modulating its effect, reinforcing its role in heading regulation. These findings are conceptually integrated and visually summarized in Figure 7, which highlights the interaction between photoperiod and temperature, the associated metabolic and transcriptomic changes, and the functional role of glycerate in heading regulation.

Despite these promising findings, several limitations remain in our study. First, due to the absence of internal standards in the metabolomics analysis, absolute intracellular concentrations of glycerate could not be determined, leaving room for future research utilizing targeted quantification approaches. Second, while we observed consistent correlations between glycerate accumulation and heading acceleration, functional validation through genome editing of glycerate translocator genes and key enzymes involved in glycerate metabolism would provide more definitive mechanistic insights. Additionally, the applied concentrations of exogenous glycerate were referenced from studies on other organic acids, and future research should refine these concentrations based on glycerate‐specific uptake and metabolic flux analyses.

Although this study successfully demonstrated the relationship between glycerate and heading responses under well‐defined temperature and photoperiod conditions, addressing these limitations will be crucial to establish a causal link and further elucidate the role of glycerate in floral transition. In particular, genetic modification approaches targeting key enzymes involved in glycerate synthesis and degradation would help clarify its precise regulatory role in floral transition. Moreover, integrating such functional approaches with controlled environmental studies could provide deeper insights into how glycerate metabolism interacts with photoperiod and temperature cues at the molecular level.

These results provide new insights into the metabolic basis of heading regulation in rice, emphasizing the need for further research on the molecular mechanisms linking photosynthesis, photorespiration, glycerate, and serine metabolism to flowering pathways. Understanding these metabolic controls could contribute to the further development of climate‐resilient rice varieties with optimized heading times for improved agricultural productivity.

## Author Contributions

**Hyeon‐Seok Lee:** conceptualization, supervision, design of experiments, gene expression analysis and field experiments, data analysis, writing – original draft preparation. **Ji‐young Shon:** conceptualization, supervision. **So‐Hye Jo:** gene expression analysis and field experiments, data analysis. **Ju‐Hee Kim:** data analysis, writing – original draft preparation. **Seo‐Yeong Yang:** data analysis. **Jae‐Kyeong Baek:** data analysis. **Yeong‐Seo Song:** data analysis. **Nam‐Jin Chung:** conceptualization, supervision. All authors discussed the results and contributed to the paper.

## Conflicts of Interest

The authors declare no conflicts of interest.

## Supporting information


**Figure S1.** Controlled environment facility at the national institute of crop science.
**Figure S2.** Sampling locations in rice plants for metabolite and transcriptome analysis.
**Figure S3.** RNA‐Sequencing analysis information.
**Figure S4.** Time‐course expression patterns of photoperiod‐responsive genes (Hd3a, RFT1, Ehd1, COL15, Ghd7) under varying temperature and photoperiod conditions at 1, 2, 4, 7, and 10 days after treatment.
**Figure S5.** Analysis of transcriptome characteristics according to temperature and day‐length treatment through RNA‐Seq.
**Figure S6.** Gas chromatograms of the extracts of rice leaves according to temperature and day‐length treatment.
**Figure S7.** Heatmaps of correlation of values of metabolites according to temperature and day‐length treatment.
**Table S1.** List of *Oryza sativa* primer sequences used for qRT‐PCR.

**Table S2.** Analysis of gene ontology (GO) according to temperature and day‐length conditions in leaves through RNA‐Seq analysis.

**Table S3.** Analysis of gene ontology (GO) according to temperature and day‐length conditions in the stem (lower part) through RNA‐Seq analysis.

**Table S4.** Analysis of gene ontology (GO) according to temperature and day‐length conditions in the stem (lower part) versus leaves through RNA‐Seq analysis.

**Table S5.** Clustering using differentially expressed genes (DEGs) according to temperature and day‐length conditions in leaves through RNA‐Seq analysis.

**Table S6.** Clustering using differentially expressed genes (DEGs) according to temperature and day‐length conditions in the stem (lower part) through RNA‐Seq analysis.

**Table S7.** Clustering using differentially expressed genes (DEGs) according to temperature and day‐length conditions in the stem (lower part) versus leaves through RNA‐Seq analysis.

**Table S8.** KEGG pathway enrichment analysis of upregulated and downregulated DEGs under different day‐length and temperature conditions in the leaf through RNA‐Seq analysis.

**Table S9.** Changes in the gene expression patterns related to photosynthesis in the KEGG pathway using differentially expressed genes (DEGs) responded to temperature and day‐length conditions in the leaf through RNA‐Seq analysis.

**Table S10.** Changes in the gene expression patterns related to glycolysis metabolism in the KEGG pathway using DEGs responded to temperature and day‐length conditions in leaf, stem (lower part), and stem (lower part) versus leaf through RNA‐Seq analysis.

**Table S11.** Changes in the gene expression patterns related to glycine, serine, and threonine metabolism in the KEGG pathway using DEGs that responded to temperature and day‐length conditions in leaves, stem (lower part), and stem (lower part) versus leaves through RNA‐Seq analysis.

## Data Availability

All data and analyses are presented in the manuscript or Supporting Information. The source data for Table 1, Figures 1, 2, 3, 4, 5, 6, 7, Tables [Link], [Link], [Link], [Link], [Link], [Link], [Link], [Link], [Link], [Link], [Link], and Figures S1–S7 are provided as Source Data files.
