# Supplementary material for: Temperature–Photoperiod Interaction in Rice Phenology for Climate Adaptation: Insights Into Glycerate‐Associated Metabolic Responses
Source: Physiol Plant. 2025 Jul 8;177(4):e70368. doi: 10.1111/ppl.70368 (PMC12238749; doi:10.1111/ppl.70368)
Supplement: Supplementary file 1 — Figure S1. Controlled environment facility at the national institute of crop science. Figure S2. Sampling locations in rice plants for metabolite and transcriptome analysis. Figure S3. RNA‐Sequencing analysis information. Figure S4. Time‐course expression patterns of photoperiod‐responsive genes (Hd3a, RFT1, Ehd1, COL15, Ghd7) under varying temperature and photoperiod conditions at 1, 2, 4, 7, and 10 days after treatment. Figure S5. Analysis of transcriptome characteristics according to temperature and day‐length treatment through RNA‐Seq. Figure S6. Gas chromatograms of the extracts of rice leaves according to temperature and day‐length treatment. Figure S7. Heatmaps of correlation of values of metabolites according to temperature and day‐length treatment. Table S1. List of Oryza sativa primer sequences used for qRT‐PCR. [file PPL-177-e70368-s007.pdf]

# Supplementary Material

**Temperature–photoperiod interaction in rice phenology for climate adaptation: insights into glycerate-associated metabolic responses**

Hyeon-Seok Lee[a*], Ju-Hee Kim[a], So-Hye Jo[a], Seo-Yeong Yang[a], Jae-Kyeong Baek[a], Yeong-Seo Song[a], Ji-young Shon[a], Nam-Jin Chung[b]

[a]Crop Production & Physiology Division, National Institute of Crop Science, Rural Development Administration, Wanju-Gun 55365, Republic of Korea

[b]Department of Crop Science and Biotechnology, Chonbuk National university, Jeonju 54896, Republic of Korea

**[*]Corresponding author:**

Hyeon-Seok Lee

E-mail: gustjr1029@korea.kr

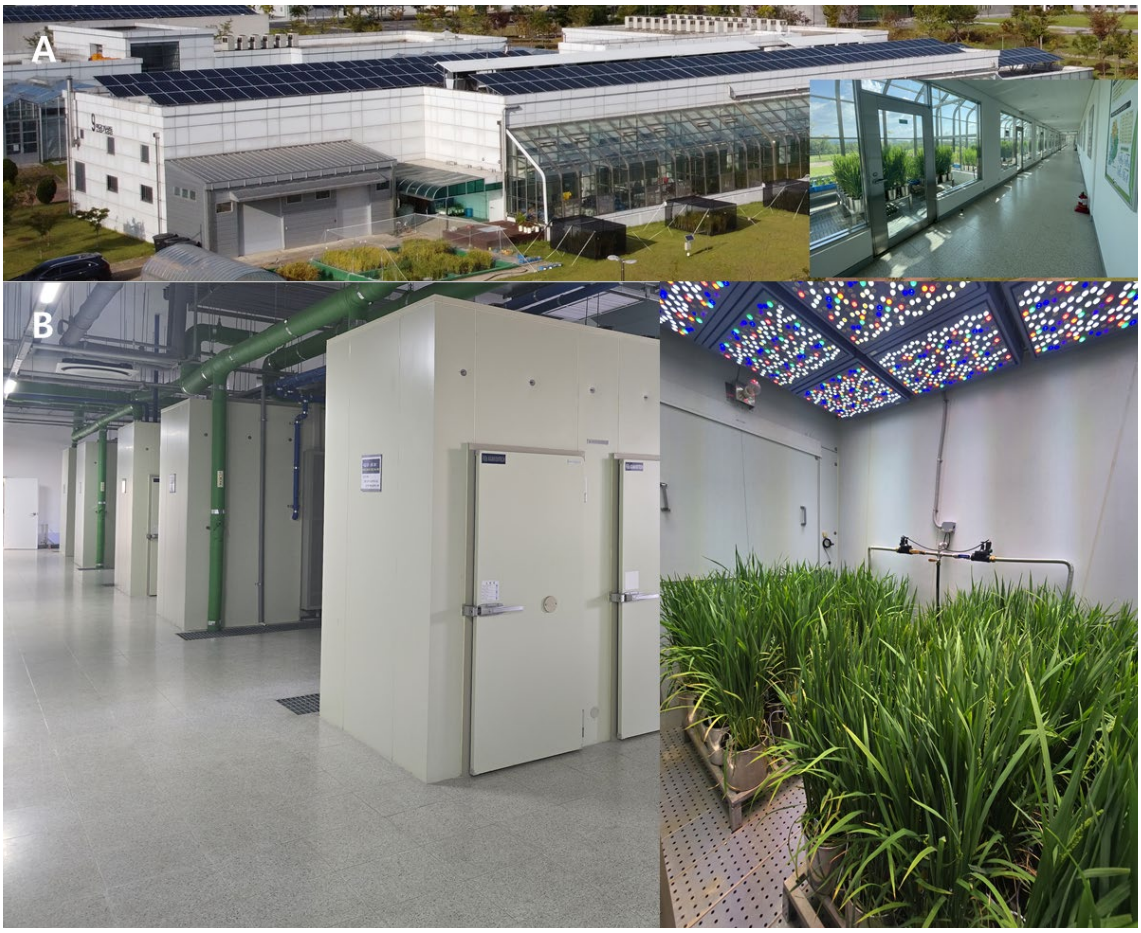


**Fig. S1. Controlled environment facility at the national institute of crop science (A), and lighting chamber where light intensity, temperature, and humidity can be artificially controlled (B).**

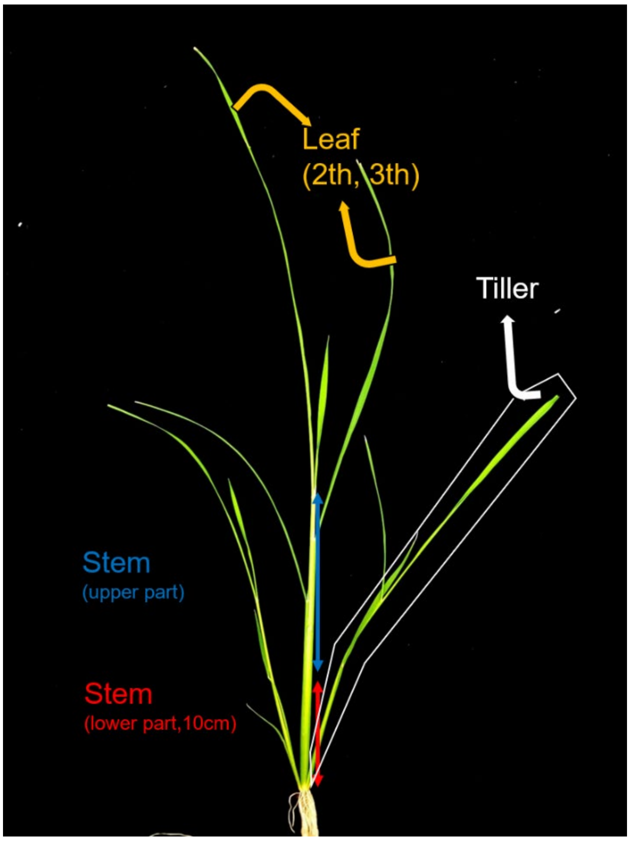


**Fig. S2. Sampling locations in rice plants for metabolite and transcriptome analysis.** Plant materials were collected at 2 days after glycerate treatment initiation, selected to capture early metabolic and transcriptional responses. The 2nd and 3rd leaves of the plant were sampled. The first tiller was sampled whole, including leaves and stems. The stems were sampled at two locations: where panicle differentiation occurs and the remaining part. The first part was sampled 10 cm from the location of the root on the stem.

A

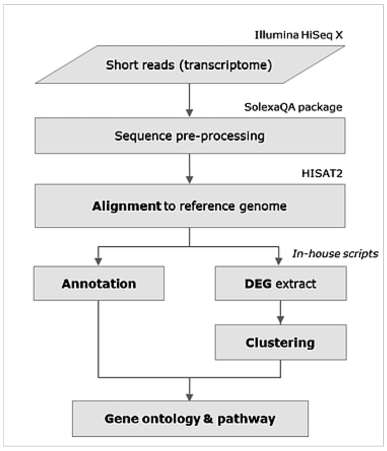


B

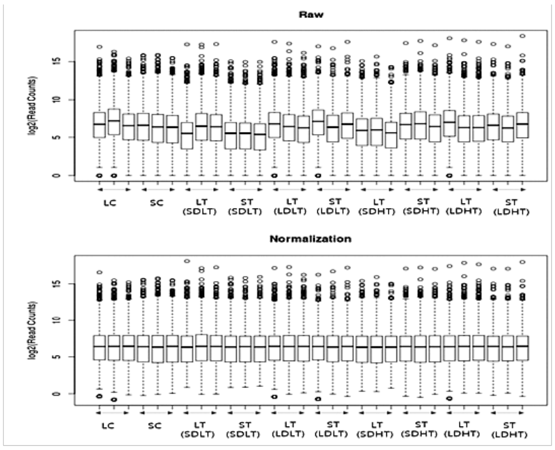


C

| Sample ID | Total reads | Aligned 0 times | | Aligned exactly 1 time | | Aligned≥ 1 times | | Aligned (discordantly or single) | | Mapping rate | |
|---|---|---|---|---|---|---|---|---|---|---|---|
| | | Reads (ea) | Percent (%) | Reads (ea) | Percent (%) | Reads (ea) | Percent (%) | Reads (ea) | Percent (%) | Reads (ea) | Percent (%) |
| L C1 | 15,159,279 | 294,135 | 1.94% | 13,136,567 | 86.66% | 404,709 | 2.67% | 1,323,867 | 8.73% | 14,865,144 | 98.06% |
| L C2 | 13,944,848 | 247,885 | 1.78% | 12,051,486 | 86.42% | 537,659 | 3.86% | 1,107,817 | 7.94% | 13,696,963 | 98.22% |
| L C3 | 14,315,313 | 244,350 | 1.71% | 12,622,016 | 88.17% | 360,115 | 2.52% | 1,088,831 | 7.61% | 14,070,963 | 98.29% |
| S C1 | 15,866,296 | 388,752 | 2.45% | 13,316,944 | 83.93% | 688,225 | 4.34% | 1,472,374 | 9.28% | 15,477,544 | 97.55% |
| S C2 | 13,650,253 | 314,561 | 2.30% | 11,593,177 | 84.93% | 579,801 | 4.25% | 1,162,713 | 8.52% | 13,335,692 | 97.70% |
| S C3 | 15,292,916 | 354,524 | 2.32% | 13,128,579 | 85.85% | 412,315 | 2.70% | 1,397,497 | 9.14% | 14,938,392 | 97.68% |
| LT (SDLT)1 | 11,605,313 | 230,201 | 1.98% | 9,986,474 | 86.05% | 418,738 | 3.61% | 969,899 | 8.36% | 11,375,112 | 98.02% |
| LT (SDLT)2 | 14,356,966 | 279,071 | 1.94% | 12,377,559 | 86.21% | 503,827 | 3.51% | 1,196,508 | 8.33% | 14,077,895 | 98.06% |
| LT (SDLT)3 | 13,986,408 | 284,958 | 2.04% | 12,073,587 | 86.32% | 377,132 | 2.70% | 1,250,730 | 8.94% | 13,701,450 | 97.96% |
| ST (SDLT)1 | 12,329,630 | 268,401 | 2.18% | 10,787,274 | 87.49% | 366,547 | 2.97% | 907,407 | 7.36% | 12,061,229 | 97.82% |
| ST (SDLT)2 | 18,326,178 | 424,595 | 2.32% | 15,405,976 | 84.07% | 822,808 | 4.49% | 1,672,798 | 9.13% | 17,901,583 | 97.68% |
| ST (SDLT)3 | 14,697,304 | 325,759 | 2.22% | 12,661,304 | 86.15% | 360,683 | 2.45% | 1,349,557 | 9.18% | 14,371,545 | 97.78% |
| LT (LDLT)1 | 12,479,384 | 215,437 | 1.73% | 10,807,054 | 86.60% | 394,041 | 3.16% | 1,062,851 | 8.52% | 12,263,947 | 98.27% |
| LT (LDLT)2 | 11,727,916 | 231,608 | 1.97% | 10,257,328 | 87.46% | 300,598 | 2.56% | 938,381 | 8.00% | 11,496,308 | 98.03% |
| LT (LDLT)3 | 13,434,611 | 259,420 | 1.93% | 11,763,905 | 87.56% | 341,489 | 2.54% | 1,069,796 | 7.96% | 13,175,191 | 98.07% |
| ST (LDLT)1 | 16,336,305 | 365,926 | 2.24% | 13,585,617 | 83.16% | 813,250 | 4.98% | 1,571,511 | 9.62% | 15,970,379 | 97.76% |
| ST (LDLT)2 | 13,337,200 | 306,439 | 2.30% | 11,259,707 | 84.42% | 550,502 | 4.13% | 1,220,551 | 9.15% | 13,030,761 | 97.70% |
| ST (LDLT)3 | 15,504,186 | 369,425 | 2.38% | 13,368,962 | 86.23% | 359,719 | 2.32% | 1,406,079 | 9.07% | 15,134,761 | 97.62% |
| LT (SDHT)1 | 12,092,866 | 254,688 | 2.11% | 10,398,144 | 85.99% | 433,898 | 3.59% | 1,006,135 | 8.32% | 11,838,178 | 97.89% |
| LT (SDHT)2 | 13,235,767 | 243,392 | 1.84% | 11,345,590 | 85.72% | 505,717 | 3.82% | 1,141,067 | 8.62% | 12,992,375 | 98.16% |
| LT (SDHT)3 | 15,215,343 | 307,741 | 2.02% | 13,360,036 | 87.81% | 427,650 | 2.81% | 1,119,916 | 7.36% | 14,907,602 | 97.98% |
| ST (SDHT)1 | 17,350,928 | 388,030 | 2.24% | 14,876,264 | 85.74% | 535,811 | 3.09% | 1,550,822 | 8.94% | 16,962,898 | 97.76% |
| ST (SDHT)2 | 13,672,018 | 276,802 | 2.02% | 11,775,057 | 86.13% | 464,345 | 3.40% | 1,155,813 | 8.45% | 13,395,216 | 97.98% |
| ST (SDHT)3 | 14,367,707 | 295,689 | 2.06% | 12,495,274 | 86.97% | 380,661 | 2.65% | 1,196,083 | 8.32% | 14,072,018 | 97.94% |
| LT (LDHT)1 | 14,308,758 | 295,777 | 2.07% | 12,341,841 | 86.25% | 478,146 | 3.34% | 1,192,993 | 8.34% | 14,012,981 | 97.93% |
| LT (LDHT)2 | 14,757,361 | 288,621 | 1.96% | 12,734,910 | 86.30% | 519,055 | 3.52% | 1,214,775 | 8.23% | 14,468,740 | 98.04% |
| LT (LDHT)3 | 14,833,375 | 280,810 | 1.89% | 12,949,264 | 87.30% | 385,692 | 2.60% | 1,217,609 | 8.21% | 14,552,565 | 98.11% |
| ST (LDHT)1 | 12,424,584 | 271,315 | 2.18% | 10,469,078 | 84.26% | 702,669 | 5.66% | 981,521 | 7.90% | 12,153,269 | 97.82% |
| ST (LDHT)2 | 13,992,417 | 296,656 | 2.12% | 11,937,900 | 85.32% | 588,324 | 4.20% | 1,169,536 | 8.36% | 13,695,761 | 97.88% |
| ST (LDHT)3 | 13,603,254 | 289,915 | 2.13% | 11,784,538 | 86.63% | 322,834 | 2.37% | 1,205,966 | 8.87% | 13,313,339 | 97.87% |
| Total | 426,204,684 | 8,894,883 | 2.08% | 366,651,412 | 86.07% | 14,336,960 | 3.36% | 1,210,713 | 8.49% | 13,910,327 | 97.92% |

**Fig. S3. RNA-Sequencing analysis information. a.** RNA- Sequencing data analysis workflow. **b.** Normalization of expression values of genes measured through read mapping using DESeq. **c.** Statistics of reads mapping to reference genome. LC: Leaves control (before treatment), SC: Stem control, LT: Leaves treatment (2 d after treatment), ST: Stem treatment.

Numbers represent repetition. The expression value was calculated by mapping the trimmed read to the reference genome sequence. Mapping was performed using HISAT software.

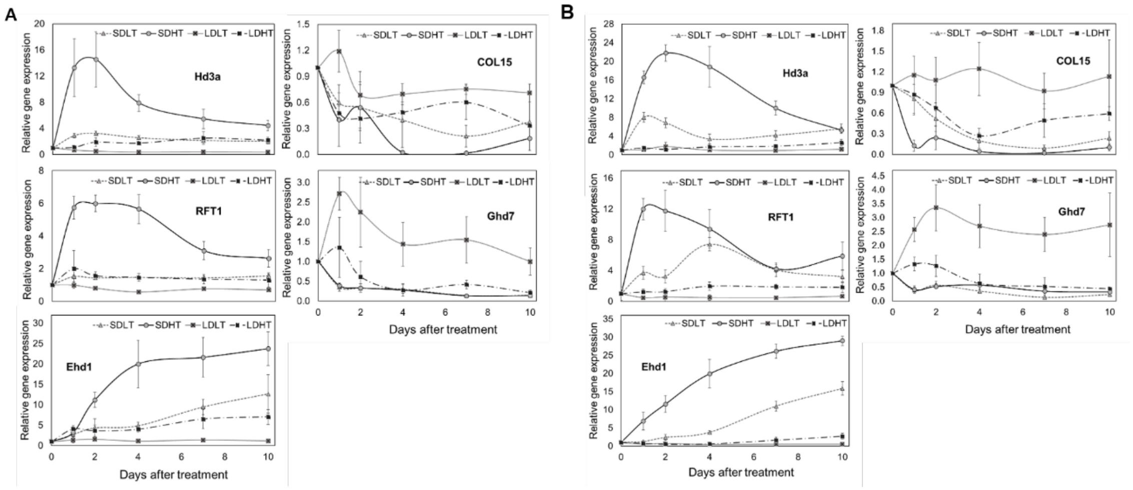


**Fig. S4. Time-course expression patterns of photoperiod-responsive genes (Hd3a, RFT1, Ehd1, COL15, Ghd7) under different temperature and photoperiod conditions at 1, 2, 4, 7, and 10 days after treatment.** (A) Odae. (B) Saenuri. Error bars indicate standard error (SE). Three biological replicates per condition.

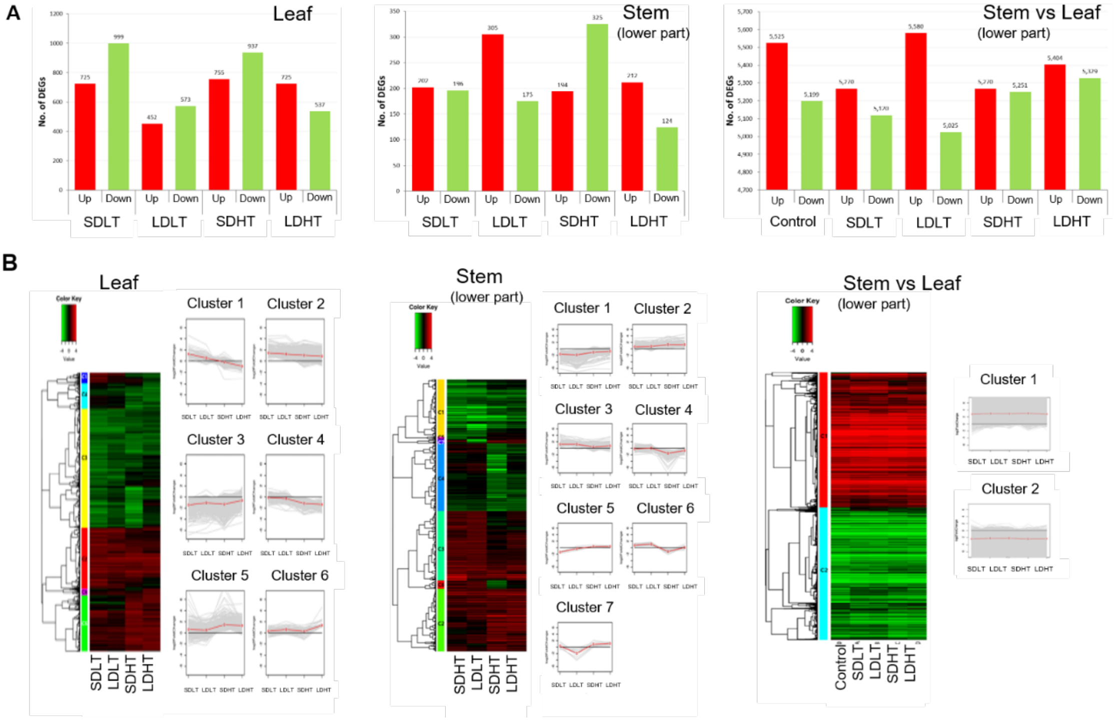


**Fig. S5. Analysis of transcriptome characteristics according to temperature and day-length treatment through RNA-Seq. (A)** Changes in the number of differentially expressed genes (DEGs) according to temperature and day-length treatment in rice leaf, stem (lower part) and stem versus leaf. **(B)** Hierarchical clustering analysis (Heat map and line plot) of differentially expressed genes according to temperature and day-length treatment in rice leaf, stem (lower part) and stem versus leaf.

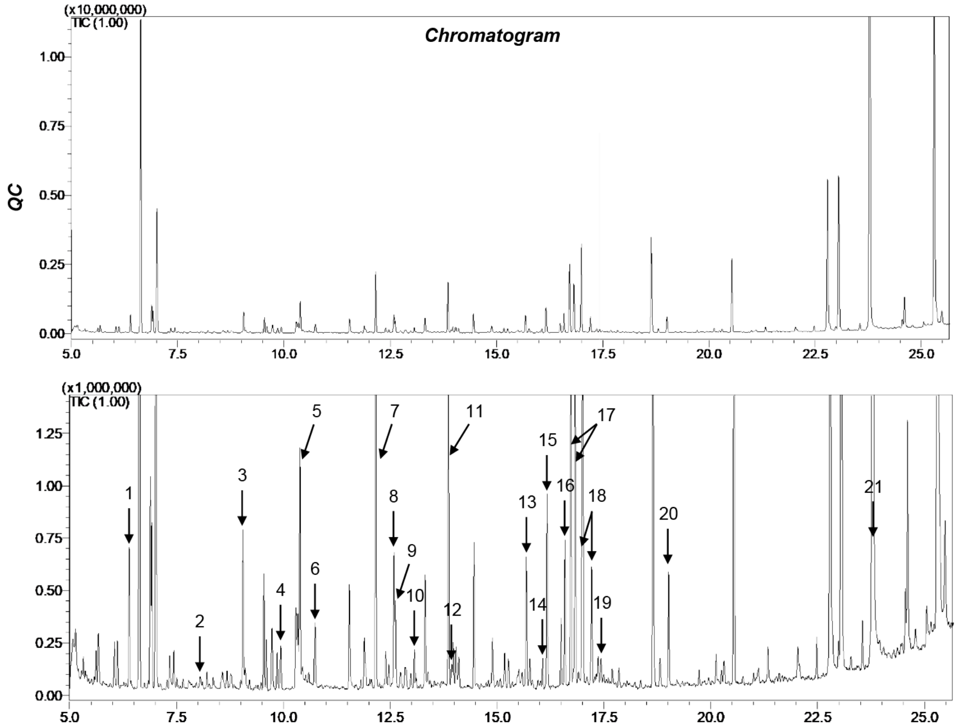


1, alanine; 2, valine; 3, phosphoric acid; 4, glyceric acid; 5, serine; 6, threonine; 7, malic acid; 8, aspartic acid; 9, oxoproline; 10, threonic acid; 11, glutamic acid; 12, asparagine 4TMS; 13, glutamine; 14, shikimic acid; 15, citric acid; 16, quinic acid; 17, fructose; 18, glucose; 19, sorbitol; 20, myo-inositol; 21, sucrose

**Fig. S6. Gas chromatograms of the extracts of rice leaves according to temperature and day-length treatment.** For GC-MS analysis, all dried samples were dissolved in methoxyamine hydrochloride in pyridine. The methoxylated samples were derivatized using N,O-bis (trimethylsilyl) trifluoroacetamide. The plant extracts were analyzed using a GC-2010 plus system equipped with a DB-5 ms capillary column. The eluents were detected using a GCMS-TQ 8030 MS (Shimadzu) system with electron ionization. The data were monitored and collected in the full-scan mode in the mass range of m/z 45–550. The QC sample was analyzed once for every 5 samples.

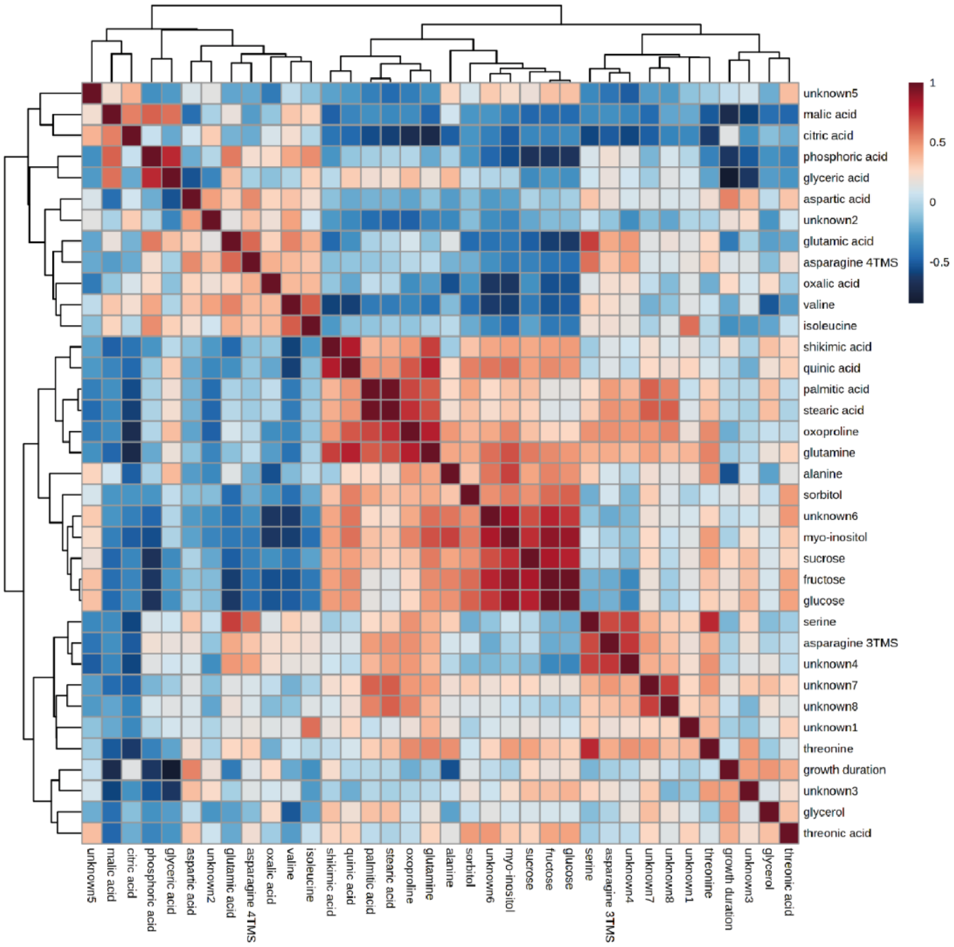


**Fig. S7. Heatmaps of correlation of values of metabolites according to temperature and day-length treatment.** Heatmaps based on Pearson Correlation Coefficient. Rows and columns are arranged by hierarchical clustering to reveal each metabolite's relationship. Red indicates positive correlations, and blue indicates negative correlations.

**Table S1.** List of primer sequences of *Oryza sativa* used for qRT-PCR.

| S. No. | Genes | Primer sequence 5'-3' |
|---|---|---|
| 1 | ***OsUBQ5-F***<br>***OsUBQ5-R*** | CCCTTGCCGACTATAACATCC<br>CAACACAACCATGACACGATG |
| 2 | ***Hd3a- F***<br>***Hd3a- R*** | GCTCACTATCATCATCCAGCATG<br>CCTTGCTCAGCTATTTAATTGCAT |
| 3 | ***RFT1-F***<br>***RFT1-R*** | TCCGAGCCCAAGCAACCCTAAC<br>AGTTCCTGGTGCTGAAGTTCTG |
| 4 | ***Ehd1-F***<br>***Ehd1-R*** | CGACAAAACACAAGACCACCCT<br>CCTGTTTGTCTGAATCCCATCG |
| 5 | ***COL15-F***<br>***COL15-R*** | GGAGATTTCTTCTGGGACAACG<br>TGTTGCTCCTGGTGCTTGG |
| 6 | ***Ghd7-F***<br>***Ghd7-R*** | AGAGGAAGAAGAGGTGCTAC<br>GACATAGGTGGATGGCGGTG |

**In separate excel files:**

**Supplementary Table 2.** Analysis of gene ontology (GO) according to temperature and day-length conditions in leaf through RNA-Seq analysis

**Supplementary Table 3.** Analysis of gene ontology (GO) according to temperature and day-length conditions in the stem (lower part) through RNA-Seq analysis

**Supplementary Table 4.** Analysis of gene ontology (GO) according to temperature and day length conditions in the stem (lower part) vs leaf through RNA-Seq analysis

**Supplementary Table 5.** Clustering using differentially expressed genes (DEGs) according to temperature and day-length conditions in leaf through RNA-Seq analysis

**Supplementary Table 6.** Clustering using differentially expressed genes (DEGs) according to temperature and day-length conditions in the stem (lower part) through RNA-Seq analysis

**Supplementary Table 7.** Clustering using differentially expressed genes (DEGs) according to temperature and day-length conditions in the stem (lower part) vs leaf through RNA-Seq analysis

**Supplementary Table 8.** KEGG pathway enrichment analysis of upregulated and downregulated DEGs under different day-length and temperature conditions in the leaf through RNA-Seq analysis.

**Supplementary Table 9.** Changes in the gene expression patterns related to photosynthesis in the KEGG pathway using differentially expressed genes (DEGs) responded to temperature and day-length conditions in the leaf through RNA-Seq analysis

**Supplementary Table 10.** Changes in the gene expression patterns related to glycolysis metabolism in the KEGG pathway using DEGs responded to temperature and day-length conditions in leaf, stem (lower part), and stem (lower part) vs leaf through RNA-Seq analysis

**Supplementary Table 11.** Changes in the gene expression patterns related to glycine, serine, and threonine metabolism in the KEGG pathway using DEGs responded to temperature and day-length conditions in leaf, stem (lower part), and stem (lower part) vs leaf through RNA-Seq analysis
